# Search for new phenomena in different-flavour high-mass dilepton final states in *pp* collisions at $\sqrt{s}=13$ Tev with the ATLAS detector

**DOI:** 10.1140/epjc/s10052-016-4385-1

**Published:** 2016-10-04

**Authors:** M. Aaboud, G. Aad, B. Abbott, J. Abdallah, O. Abdinov, B. Abeloos, R. Aben, O. S. AbouZeid, N. L. Abraham, H. Abramowicz, H. Abreu, R. Abreu, Y. Abulaiti, B. S. Acharya, S. Adachi, L. Adamczyk, D. L. Adams, J. Adelman, S. Adomeit, T. Adye, A. A. Affolder, T. Agatonovic-Jovin, J. Agricola, J. A. Aguilar-Saavedra, S. P. Ahlen, F. Ahmadov, G. Aielli, H. Akerstedt, T. P. A. Åkesson, A. V. Akimov, G. L. Alberghi, J. Albert, S. Albrand, M. J. Alconada Verzini, M. Aleksa, I. N. Aleksandrov, C. Alexa, G. Alexander, T. Alexopoulos, M. Alhroob, B. Ali, M. Aliev, G. Alimonti, J. Alison, S. P. Alkire, B. M. M. Allbrooke, B. W. Allen, P. P. Allport, A. Aloisio, A. Alonso, F. Alonso, C. Alpigiani, A. A. Alshehri, M. Alstaty, B. Alvarez Gonzalez, D. Álvarez Piqueras, M. G. Alviggi, B. T. Amadio, K. Amako, Y. Amaral Coutinho, C. Amelung, D. Amidei, S. P. Amor Dos Santos, A. Amorim, S. Amoroso, G. Amundsen, C. Anastopoulos, L. S. Ancu, N. Andari, T. Andeen, C. F. Anders, G. Anders, J. K. Anders, K. J. Anderson, A. Andreazza, V. Andrei, S. Angelidakis, I. Angelozzi, P. Anger, A. Angerami, F. Anghinolfi, A. V. Anisenkov, N. Anjos, A. Annovi, C. Antel, M. Antonelli, A. Antonov, F. Anulli, M. Aoki, L. Aperio Bella, G. Arabidze, Y. Arai, J. P. Araque, A. T. H. Arce, F. A. Arduh, J-F. Arguin, S. Argyropoulos, M. Arik, A. J. Armbruster, L. J. Armitage, O. Arnaez, H. Arnold, M. Arratia, O. Arslan, A. Artamonov, G. Artoni, S. Artz, S. Asai, N. Asbah, A. Ashkenazi, B. Åsman, L. Asquith, K. Assamagan, R. Astalos, M. Atkinson, N. B. Atlay, K. Augsten, G. Avolio, B. Axen, M. K. Ayoub, G. Azuelos, M. A. Baak, A. E. Baas, M. J. Baca, H. Bachacou, K. Bachas, M. Backes, M. Backhaus, P. Bagiacchi, P. Bagnaia, Y. Bai, J. T. Baines, O. K. Baker, E. M. Baldin, P. Balek, T. Balestri, F. Balli, W. K. Balunas, E. Banas, Sw. Banerjee, A. A. E. Bannoura, L. Barak, E. L. Barberio, D. Barberis, M. Barbero, T. Barillari, M-S Barisits, T. Barklow, N. Barlow, S. L. Barnes, B. M. Barnett, R. M. Barnett, Z. Barnovska-Blenessy, A. Baroncelli, G. Barone, A. J. Barr, L. Barranco Navarro, F. Barreiro, J. Barreiro Guimarães da Costa, R. Bartoldus, A. E. Barton, P. Bartos, A. Basalaev, A. Bassalat, R. L. Bates, S. J. Batista, J. R. Batley, M. Battaglia, M. Bauce, F. Bauer, H. S. Bawa, J. B. Beacham, M. D. Beattie, T. Beau, P. H. Beauchemin, P. Bechtle, H. P. Beck, K. Becker, M. Becker, M. Beckingham, C. Becot, A. J. Beddall, A. Beddall, V. A. Bednyakov, M. Bedognetti, C. P. Bee, L. J. Beemster, T. A. Beermann, M. Begel, J. K. Behr, C. Belanger-Champagne, A. S. Bell, G. Bella, L. Bellagamba, A. Bellerive, M. Bellomo, K. Belotskiy, O. Beltramello, N. L. Belyaev, O. Benary, D. Benchekroun, M. Bender, K. Bendtz, N. Benekos, Y. Benhammou, E. Benhar Noccioli, J. Benitez, D. P. Benjamin, J. R. Bensinger, S. Bentvelsen, L. Beresford, M. Beretta, D. Berge, E. Bergeaas Kuutmann, N. Berger, J. Beringer, S. Berlendis, N. R. Bernard, C. Bernius, F. U. Bernlochner, T. Berry, P. Berta, C. Bertella, G. Bertoli, F. Bertolucci, I. A. Bertram, C. Bertsche, D. Bertsche, G. J. Besjes, O. Bessidskaia Bylund, M. Bessner, N. Besson, C. Betancourt, A. Bethani, S. Bethke, A. J. Bevan, R. M. Bianchi, L. Bianchini, M. Bianco, O. Biebel, D. Biedermann, R. Bielski, N. V. Biesuz, M. Biglietti, J. Bilbao De Mendizabal, T. R. V. Billoud, H. Bilokon, M. Bindi, S. Binet, A. Bingul, C. Bini, S. Biondi, T. Bisanz, D. M. Bjergaard, C. W. Black, J. E. Black, K. M. Black, D. Blackburn, R. E. Blair, J. -B. Blanchard, T. Blazek, I. Bloch, C. Blocker, A. Blue, W. Blum, U. Blumenschein, S. Blunier, G. J. Bobbink, V. S. Bobrovnikov, S. S. Bocchetta, A. Bocci, C. Bock, M. Boehler, D. Boerner, J. A. Bogaerts, D. Bogavac, A. G. Bogdanchikov, C. Bohm, V. Boisvert, P. Bokan, T. Bold, A. S. Boldyrev, M. Bomben, M. Bona, M. Boonekamp, A. Borisov, G. Borissov, J. Bortfeldt, D. Bortoletto, V. Bortolotto, K. Bos, D. Boscherini, M. Bosman, J. D. Bossio Sola, J. Boudreau, J. Bouffard, E. V. Bouhova-Thacker, D. Boumediene, C. Bourdarios, S. K. Boutle, A. Boveia, J. Boyd, I. R. Boyko, J. Bracinik, A. Brandt, G. Brandt, O. Brandt, U. Bratzler, B. Brau, J. E. Brau, W. D. Breaden Madden, K. Brendlinger, A. J. Brennan, L. Brenner, R. Brenner, S. Bressler, T. M. Bristow, D. Britton, D. Britzger, F. M. Brochu, I. Brock, R. Brock, G. Brooijmans, T. Brooks, W. K. Brooks, J. Brosamer, E. Brost, J.H Broughton, P. A. Bruckman de Renstrom, D. Bruncko, R. Bruneliere, A. Bruni, G. Bruni, L. S. Bruni, BH Brunt, M. Bruschi, N. Bruscino, P. Bryant, L. Bryngemark, T. Buanes, Q. Buat, P. Buchholz, A. G. Buckley, I. A. Budagov, F. Buehrer, M. K. Bugge, O. Bulekov, D. Bullock, H. Burckhart, S. Burdin, C. D. Burgard, B. Burghgrave, K. Burka, S. Burke, I. Burmeister, J. T. P. Burr, E. Busato, D. Büscher, V. Büscher, P. Bussey, J. M. Butler, C. M. Buttar, J. M. Butterworth, P. Butti, W. Buttinger, A. Buzatu, A. R. Buzykaev, S. Cabrera Urbán, D. Caforio, V. M. Cairo, O. Cakir, N. Calace, P. Calafiura, A. Calandri, G. Calderini, P. Calfayan, G. Callea, L. P. Caloba, S. Calvente Lopez, D. Calvet, S. Calvet, T. P. Calvet, R. Camacho Toro, S. Camarda, P. Camarri, D. Cameron, R. Caminal Armadans, C. Camincher, S. Campana, M. Campanelli, A. Camplani, A. Campoverde, V. Canale, A. Canepa, M. Cano Bret, J. Cantero, T. Cao, M. D. M. Capeans Garrido, I. Caprini, M. Caprini, M. Capua, R. M. Carbone, R. Cardarelli, F. Cardillo, I. Carli, T. Carli, G. Carlino, L. Carminati, S. Caron, E. Carquin, G. D. Carrillo-Montoya, J. R. Carter, J. Carvalho, D. Casadei, M. P. Casado, M. Casolino, D. W. Casper, E. Castaneda-Miranda, R. Castelijn, A. Castelli, V. Castillo Gimenez, N. F. Castro, A. Catinaccio, J. R. Catmore, A. Cattai, J. Caudron, V. Cavaliere, E. Cavallaro, D. Cavalli, M. Cavalli-Sforza, V. Cavasinni, F. Ceradini, L. Cerda Alberich, B. C. Cerio, A. S. Cerqueira, A. Cerri, L. Cerrito, F. Cerutti, M. Cerv, A. Cervelli, S. A. Cetin, A. Chafaq, D. Chakraborty, S. K. Chan, Y. L. Chan, P. Chang, J. D. Chapman, D. G. Charlton, A. Chatterjee, C. C. Chau, C. A. Chavez Barajas, S. Che, S. Cheatham, A. Chegwidden, S. Chekanov, S. V. Chekulaev, G. A. Chelkov, M. A. Chelstowska, C. Chen, H. Chen, K. Chen, S. Chen, S. Chen, X. Chen, Y. Chen, H. C. Cheng, H. J Cheng, Y. Cheng, A. Cheplakov, E. Cheremushkina, R. Cherkaoui El Moursli, V. Chernyatin, E. Cheu, L. Chevalier, V. Chiarella, G. Chiarelli, G. Chiodini, A. S. Chisholm, A. Chitan, M. V. Chizhov, K. Choi, A. R. Chomont, S. Chouridou, B. K. B. Chow, V. Christodoulou, D. Chromek-Burckhart, J. Chudoba, A. J. Chuinard, J. J. Chwastowski, L. Chytka, G. Ciapetti, A. K. Ciftci, D. Cinca, V. Cindro, I. A. Cioara, C. Ciocca, A. Ciocio, F. Cirotto, Z. H. Citron, M. Citterio, M. Ciubancan, A. Clark, B. L. Clark, M. R. Clark, P. J. Clark, R. N. Clarke, C. Clement, Y. Coadou, M. Cobal, A. Coccaro, J. Cochran, L. Colasurdo, B. Cole, A. P. Colijn, J. Collot, T. Colombo, G. Compostella, P. Conde Muiño, E. Coniavitis, S. H. Connell, I. A. Connelly, V. Consorti, S. Constantinescu, G. Conti, F. Conventi, M. Cooke, B. D. Cooper, A. M. Cooper-Sarkar, K. J. R. Cormier, T. Cornelissen, M. Corradi, F. Corriveau, A. Corso-Radu, A. Cortes-Gonzalez, G. Cortiana, G. Costa, M. J. Costa, D. Costanzo, G. Cottin, G. Cowan, B. E. Cox, K. Cranmer, S. J. Crawley, G. Cree, S. Crépé-Renaudin, F. Crescioli, W. A. Cribbs, M. Crispin Ortuzar, M. Cristinziani, V. Croft, G. Crosetti, A. Cueto, T. Cuhadar Donszelmann, J. Cummings, M. Curatolo, J. Cúth, H. Czirr, P. Czodrowski, G. D’amen, S. D’Auria, M. D’Onofrio, M. J. Da Cunha Sargedas De Sousa, C. Da Via, W. Dabrowski, T. Dado, T. Dai, O. Dale, F. Dallaire, C. Dallapiccola, M. Dam, J. R. Dandoy, N. P. Dang, A. C. Daniells, N. S. Dann, M. Danninger, M. Dano Hoffmann, V. Dao, G. Darbo, S. Darmora, J. Dassoulas, A. Dattagupta, W. Davey, C. David, T. Davidek, M. Davies, P. Davison, E. Dawe, I. Dawson, K. De, R. de Asmundis, A. De Benedetti, S. De Castro, S. De Cecco, N. De Groot, P. de Jong, H. De la Torre, F. De Lorenzi, A. De Maria, D. De Pedis, A. De Salvo, U. De Sanctis, A. De Santo, J. B. De Vivie De Regie, W. J. Dearnaley, R. Debbe, C. Debenedetti, D. V. Dedovich, N. Dehghanian, I. Deigaard, M. Del Gaudio, J. Del Peso, T. Del Prete, D. Delgove, F. Deliot, C. M. Delitzsch, A. Dell’Acqua, L. Dell’Asta, M. Dell’Orso, M. Della Pietra, D. della Volpe, M. Delmastro, P. A. Delsart, D. A. DeMarco, S. Demers, M. Demichev, A. Demilly, S. P. Denisov, D. Denysiuk, D. Derendarz, J. E. Derkaoui, F. Derue, P. Dervan, K. Desch, C. Deterre, K. Dette, P. O. Deviveiros, A. Dewhurst, S. Dhaliwal, A. Di Ciaccio, L. Di Ciaccio, W. K. Di Clemente, C. Di Donato, A. Di Girolamo, B. Di Girolamo, B. Di Micco, R. Di Nardo, A. Di Simone, R. Di Sipio, D. Di Valentino, C. Diaconu, M. Diamond, F. A. Dias, M. A. Diaz, E. B. Diehl, J. Dietrich, S. Díez Cornell, A. Dimitrievska, J. Dingfelder, P. Dita, S. Dita, F. Dittus, F. Djama, T. Djobava, J. I. Djuvsland, M. A. B. do Vale, D. Dobos, M. Dobre, C. Doglioni, J. Dolejsi, Z. Dolezal, M. Donadelli, S. Donati, P. Dondero, J. Donini, J. Dopke, A. Doria, M. T. Dova, A. T. Doyle, E. Drechsler, M. Dris, Y. Du, J. Duarte-Campderros, E. Duchovni, G. Duckeck, O. A. Ducu, D. Duda, A. Dudarev, A. Chr. Dudder, E. M. Duffield, L. Duflot, M. Dührssen, M. Dumancic, M. Dunford, H. Duran Yildiz, M. Düren, A. Durglishvili, D. Duschinger, B. Dutta, M. Dyndal, C. Eckardt, K. M. Ecker, R. C. Edgar, N. C. Edwards, T. Eifert, G. Eigen, K. Einsweiler, T. Ekelof, M. El Kacimi, V. Ellajosyula, M. Ellert, S. Elles, F. Ellinghaus, A. A. Elliot, N. Ellis, J. Elmsheuser, M. Elsing, D. Emeliyanov, Y. Enari, O. C. Endner, J. S. Ennis, J. Erdmann, A. Ereditato, G. Ernis, J. Ernst, M. Ernst, S. Errede, E. Ertel, M. Escalier, H. Esch, C. Escobar, B. Esposito, A. I. Etienvre, E. Etzion, H. Evans, A. Ezhilov, M. Ezzi, F. Fabbri, L. Fabbri, G. Facini, R. M. Fakhrutdinov, S. Falciano, R. J. Falla, J. Faltova, Y. Fang, M. Fanti, A. Farbin, A. Farilla, C. Farina, E. M. Farina, T. Farooque, S. Farrell, S. M. Farrington, P. Farthouat, F. Fassi, P. Fassnacht, D. Fassouliotis, M. Faucci Giannelli, A. Favareto, W. J. Fawcett, L. Fayard, O. L. Fedin, W. Fedorko, S. Feigl, L. Feligioni, C. Feng, E. J. Feng, H. Feng, A. B. Fenyuk, L. Feremenga, P. Fernandez Martinez, S. Fernandez Perez, J. Ferrando, A. Ferrari, P. Ferrari, R. Ferrari, D. E. Ferreira de Lima, A. Ferrer, D. Ferrere, C. Ferretti, A. Ferretto Parodi, F. Fiedler, A. Filipčič, M. Filipuzzi, F. Filthaut, M. Fincke-Keeler, K. D. Finelli, M. C. N. Fiolhais, L. Fiorini, A. Firan, A. Fischer, C. Fischer, J. Fischer, W. C. Fisher, N. Flaschel, I. Fleck, P. Fleischmann, G. T. Fletcher, R. R. M. Fletcher, T. Flick, L. R. Flores Castillo, M. J. Flowerdew, G. T. Forcolin, A. Formica, A. Forti, A. G. Foster, D. Fournier, H. Fox, S. Fracchia, P. Francavilla, M. Franchini, D. Francis, L. Franconi, M. Franklin, M. Frate, M. Fraternali, D. Freeborn, S. M. Fressard-Batraneanu, F. Friedrich, D. Froidevaux, J. A. Frost, C. Fukunaga, E. Fullana Torregrosa, T. Fusayasu, J. Fuster, C. Gabaldon, O. Gabizon, A. Gabrielli, A. Gabrielli, G. P. Gach, S. Gadatsch, S. Gadomski, G. Gagliardi, L. G. Gagnon, P. Gagnon, C. Galea, B. Galhardo, E. J. Gallas, B. J. Gallop, P. Gallus, G. Galster, K. K. Gan, J. Gao, Y. Gao, Y. S. Gao, F. M. Garay Walls, C. García, J. E. García Navarro, M. Garcia-Sciveres, R. W. Gardner, N. Garelli, V. Garonne, A. Gascon Bravo, K. Gasnikova, C. Gatti, A. Gaudiello, G. Gaudio, L. Gauthier, I. L. Gavrilenko, C. Gay, G. Gaycken, E. N. Gazis, Z. Gecse, C. N. P. Gee, Ch. Geich-Gimbel, M. Geisen, M. P. Geisler, K. Gellerstedt, C. Gemme, M. H. Genest, C. Geng, S. Gentile, C. Gentsos, S. George, D. Gerbaudo, A. Gershon, S. Ghasemi, M. Ghneimat, B. Giacobbe, S. Giagu, P. Giannetti, B. Gibbard, S. M. Gibson, M. Gignac, M. Gilchriese, T. P. S. Gillam, D. Gillberg, G. Gilles, D. M. Gingrich, N. Giokaris, M. P. Giordani, F. M. Giorgi, F. M. Giorgi, P. F. Giraud, P. Giromini, D. Giugni, F. Giuli, C. Giuliani, M. Giulini, B. K. Gjelsten, S. Gkaitatzis, I. Gkialas, E. L. Gkougkousis, L. K. Gladilin, C. Glasman, J. Glatzer, P. C. F. Glaysher, A. Glazov, M. Goblirsch-Kolb, J. Godlewski, S. Goldfarb, T. Golling, D. Golubkov, A. Gomes, R. Gonçalo, J. Goncalves Pinto Firmino Da Costa, G. Gonella, L. Gonella, A. Gongadze, S. González de la Hoz, G. Gonzalez Parra, S. Gonzalez-Sevilla, L. Goossens, P. A. Gorbounov, H. A. Gordon, I. Gorelov, B. Gorini, E. Gorini, A. Gorišek, E. Gornicki, A. T. Goshaw, C. Gössling, M. I. Gostkin, C. R. Goudet, D. Goujdami, A. G. Goussiou, N. Govender, E. Gozani, L. Graber, I. Grabowska-Bold, P. O. J. Gradin, P. Grafström, J. Gramling, E. Gramstad, S. Grancagnolo, V. Gratchev, P. M. Gravila, H. M. Gray, E. Graziani, Z. D. Greenwood, C. Grefe, K. Gregersen, I. M. Gregor, P. Grenier, K. Grevtsov, J. Griffiths, A. A. Grillo, K. Grimm, S. Grinstein, Ph. Gris, J. -F. Grivaz, S. Groh, J. P. Grohs, E. Gross, J. Grosse-Knetter, G. C. Grossi, Z. J. Grout, L. Guan, W. Guan, J. Guenther, F. Guescini, D. Guest, O. Gueta, E. Guido, T. Guillemin, S. Guindon, U. Gul, C. Gumpert, J. Guo, Y. Guo, R. Gupta, S. Gupta, G. Gustavino, P. Gutierrez, N. G. Gutierrez Ortiz, C. Gutschow, C. Guyot, C. Gwenlan, C. B. Gwilliam, A. Haas, C. Haber, H. K. Hadavand, N. Haddad, A. Hadef, S. Hageböck, M. Hagihara, Z. Hajduk, H. Hakobyan, M. Haleem, J. Haley, G. Halladjian, G. D. Hallewell, K. Hamacher, P. Hamal, K. Hamano, A. Hamilton, G. N. Hamity, P. G. Hamnett, L. Han, K. Hanagaki, K. Hanawa, M. Hance, B. Haney, P. Hanke, R. Hanna, J. B. Hansen, J. D. Hansen, M. C. Hansen, P. H. Hansen, K. Hara, A. S. Hard, T. Harenberg, F. Hariri, S. Harkusha, R. D. Harrington, P. F. Harrison, F. Hartjes, N. M. Hartmann, M. Hasegawa, Y. Hasegawa, A. Hasib, S. Hassani, S. Haug, R. Hauser, L. Hauswald, M. Havranek, C. M. Hawkes, R. J. Hawkings, D. Hayakawa, D. Hayden, C. P. Hays, J. M. Hays, H. S. Hayward, S. J. Haywood, S. J. Head, T. Heck, V. Hedberg, L. Heelan, S. Heim, T. Heim, B. Heinemann, J. J. Heinrich, L. Heinrich, C. Heinz, J. Hejbal, L. Helary, S. Hellman, C. Helsens, J. Henderson, R. C. W. Henderson, Y. Heng, S. Henkelmann, A. M. Henriques Correia, S. Henrot-Versille, G. H. Herbert, H. Herde, V. Herget, Y. Hernández Jiménez, G. Herten, R. Hertenberger, L. Hervas, G. G. Hesketh, N. P. Hessey, J. W. Hetherly, R. Hickling, E. Higón-Rodriguez, E. Hill, J. C. Hill, K. H. Hiller, S. J. Hillier, I. Hinchliffe, E. Hines, R. R. Hinman, M. Hirose, D. Hirschbuehl, J. Hobbs, N. Hod, M. C. Hodgkinson, P. Hodgson, A. Hoecker, M. R. Hoeferkamp, F. Hoenig, D. Hohn, T. R. Holmes, M. Homann, T. Honda, T. M. Hong, B. H. Hooberman, W. H. Hopkins, Y. Horii, A. J. Horton, J-Y. Hostachy, S. Hou, A. Hoummada, J. Howarth, J. Hoya, M. Hrabovsky, I. Hristova, J. Hrivnac, T. Hryn’ova, A. Hrynevich, C. Hsu, P. J. Hsu, S. -C. Hsu, Q. Hu, S. Hu, Y. Huang, Z. Hubacek, F. Hubaut, F. Huegging, T. B. Huffman, E. W. Hughes, G. Hughes, M. Huhtinen, P. Huo, N. Huseynov, J. Huston, J. Huth, G. Iacobucci, G. Iakovidis, I. Ibragimov, L. Iconomidou-Fayard, E. Ideal, Z. Idrissi, P. Iengo, O. Igonkina, T. Iizawa, Y. Ikegami, M. Ikeno, Y. Ilchenko, D. Iliadis, N. Ilic, T. Ince, G. Introzzi, P. Ioannou, M. Iodice, K. Iordanidou, V. Ippolito, N. Ishijima, M. Ishino, M. Ishitsuka, R. Ishmukhametov, C. Issever, S. Istin, F. Ito, J. M. Iturbe Ponce, R. Iuppa, W. Iwanski, H. Iwasaki, J. M. Izen, V. Izzo, S. Jabbar, B. Jackson, P. Jackson, V. Jain, K. B. Jakobi, K. Jakobs, S. Jakobsen, T. Jakoubek, D. O. Jamin, D. K. Jana, R. Jansky, J. Janssen, M. Janus, G. Jarlskog, N. Javadov, T. Javůrek, F. Jeanneau, L. Jeanty, G. -Y. Jeng, D. Jennens, P. Jenni, C. Jeske, S. Jézéquel, H. Ji, J. Jia, H. Jiang, Y. Jiang, S. Jiggins, J. Jimenez Pena, S. Jin, A. Jinaru, O. Jinnouchi, H. Jivan, P. Johansson, K. A. Johns, W. J. Johnson, K. Jon-And, G. Jones, R. W. L. Jones, S. Jones, T. J. Jones, J. Jongmanns, P. M. Jorge, J. Jovicevic, X. Ju, A. Juste Rozas, M. K. Köhler, A. Kaczmarska, M. Kado, H. Kagan, M. Kagan, S. J. Kahn, T. Kaji, E. Kajomovitz, C. W. Kalderon, A. Kaluza, S. Kama, A. Kamenshchikov, N. Kanaya, S. Kaneti, L. Kanjir, V. A. Kantserov, J. Kanzaki, B. Kaplan, L. S. Kaplan, A. Kapliy, D. Kar, K. Karakostas, A. Karamaoun, N. Karastathis, M. J. Kareem, E. Karentzos, M. Karnevskiy, S. N. Karpov, Z. M. Karpova, K. Karthik, V. Kartvelishvili, A. N. Karyukhin, K. Kasahara, L. Kashif, R. D. Kass, A. Kastanas, Y. Kataoka, C. Kato, A. Katre, J. Katzy, K. Kawade, K. Kawagoe, T. Kawamoto, G. Kawamura, V. F. Kazanin, R. Keeler, R. Kehoe, J. S. Keller, J. J. Kempster, H. Keoshkerian, O. Kepka, B. P. Kerševan, S. Kersten, R. A. Keyes, M. Khader, F. Khalil-zada, A. Khanov, A. G. Kharlamov, T. Kharlamova, T. J. Khoo, V. Khovanskiy, E. Khramov, J. Khubua, S. Kido, C. R. Kilby, H. Y. Kim, S. H. Kim, Y. K. Kim, N. Kimura, O. M. Kind, B. T. King, M. King, J. Kirk, A. E. Kiryunin, T. Kishimoto, D. Kisielewska, F. Kiss, K. Kiuchi, O. Kivernyk, E. Kladiva, M. H. Klein, M. Klein, U. Klein, K. Kleinknecht, P. Klimek, A. Klimentov, R. Klingenberg, J. A. Klinger, T. Klioutchnikova, E. -E. Kluge, P. Kluit, S. Kluth, J. Knapik, E. Kneringer, E. B. F. G. Knoops, A. Knue, A. Kobayashi, D. Kobayashi, T. Kobayashi, M. Kobel, M. Kocian, P. Kodys, N. M. Koehler, T. Koffas, E. Koffeman, T. Koi, H. Kolanoski, M. Kolb, I. Koletsou, A. A. Komar, Y. Komori, T. Kondo, N. Kondrashova, K. Köneke, A. C. König, T. Kono, R. Konoplich, N. Konstantinidis, R. Kopeliansky, S. Koperny, L. Köpke, A. K. Kopp, K. Korcyl, K. Kordas, A. Korn, A. A. Korol, I. Korolkov, E. V. Korolkova, O. Kortner, S. Kortner, T. Kosek, V. V. Kostyukhin, A. Kotwal, A. Kourkoumeli-Charalampidi, C. Kourkoumelis, V. Kouskoura, A. B. Kowalewska, R. Kowalewski, T. Z. Kowalski, C. Kozakai, W. Kozanecki, A. S. Kozhin, V. A. Kramarenko, G. Kramberger, D. Krasnopevtsev, M. W. Krasny, A. Krasznahorkay, A. Kravchenko, M. Kretz, J. Kretzschmar, K. Kreutzfeldt, P. Krieger, K. Krizka, K. Kroeninger, H. Kroha, J. Kroll, J. Kroseberg, J. Krstic, U. Kruchonak, H. Krüger, N. Krumnack, M. C. Kruse, M. Kruskal, T. Kubota, H. Kucuk, S. Kuday, J. T. Kuechler, S. Kuehn, A. Kugel, F. Kuger, A. Kuhl, T. Kuhl, V. Kukhtin, R. Kukla, Y. Kulchitsky, S. Kuleshov, M. Kuna, T. Kunigo, A. Kupco, H. Kurashige, Y. A. Kurochkin, V. Kus, E. S. Kuwertz, M. Kuze, J. Kvita, T. Kwan, D. Kyriazopoulos, A. La Rosa, J. L. La Rosa Navarro, L. La Rotonda, C. Lacasta, F. Lacava, J. Lacey, H. Lacker, D. Lacour, V. R. Lacuesta, E. Ladygin, R. Lafaye, B. Laforge, T. Lagouri, S. Lai, S. Lammers, W. Lampl, E. Lançon, U. Landgraf, M. P. J. Landon, M. C. Lanfermann, V. S. Lang, J. C. Lange, A. J. Lankford, F. Lanni, K. Lantzsch, A. Lanza, S. Laplace, C. Lapoire, J. F. Laporte, T. Lari, F. Lasagni Manghi, M. Lassnig, P. Laurelli, W. Lavrijsen, A. T. Law, P. Laycock, T. Lazovich, M. Lazzaroni, B. Le, O. Le Dortz, E. Le Guirriec, E. P. Le Quilleuc, M. LeBlanc, T. LeCompte, F. Ledroit-Guillon, C. A. Lee, S. C. Lee, L. Lee, B. Lefebvre, G. Lefebvre, M. Lefebvre, F. Legger, C. Leggett, A. Lehan, G. Lehmann Miotto, X. Lei, W. A. Leight, A. G. Leister, M. A. L. Leite, R. Leitner, D. Lellouch, B. Lemmer, K. J. C. Leney, T. Lenz, B. Lenzi, R. Leone, S. Leone, C. Leonidopoulos, S. Leontsinis, G. Lerner, C. Leroy, A. A. J. Lesage, C. G. Lester, M. Levchenko, J. Levêque, D. Levin, L. J. Levinson, M. Levy, D. Lewis, A. M. Leyko, M. Leyton, B. Li, C. Li, H. Li, H. L. Li, L. Li, L. Li, Q. Li, Q. Li, S. Li, X. Li, Y. Li, Z. Liang, B. Liberti, A. Liblong, P. Lichard, K. Lie, J. Liebal, W. Liebig, A. Limosani, S. C. Lin, T. H. Lin, B. E. Lindquist, A. E. Lionti, E. Lipeles, A. Lipniacka, M. Lisovyi, T. M. Liss, A. Lister, A. M. Litke, B. Liu, D. Liu, H. Liu, H. Liu, J. Liu, J. B. Liu, K. Liu, L. Liu, M. Liu, M. Liu, Y. L. Liu, Y. Liu, M. Livan, A. Lleres, J. Llorente Merino, S. L. Lloyd, F. Lo Sterzo, E. M. Lobodzinska, P. Loch, W. S. Lockman, F. K. Loebinger, A. E. Loevschall-Jensen, K. M. Loew, A. Loginov, T. Lohse, K. Lohwasser, M. Lokajicek, B. A. Long, J. D. Long, R. E. Long, L. Longo, K. A. Looper, J. A. López, D. Lopez Mateos, B. Lopez Paredes, I. Lopez Paz, A. Lopez Solis, J. Lorenz, N. Lorenzo Martinez, M. Losada, P. J. Lösel, X. Lou, A. Lounis, J. Love, P. A. Love, H. Lu, N. Lu, H. J. Lubatti, C. Luci, A. Lucotte, C. Luedtke, F. Luehring, W. Lukas, L. Luminari, O. Lundberg, B. Lund-Jensen, P. M. Luzi, D. Lynn, R. Lysak, E. Lytken, V. Lyubushkin, H. Ma, L. L. Ma, Y. Ma, G. Maccarrone, A. Macchiolo, C. M. Macdonald, B. Maček, J. Machado Miguens, D. Madaffari, R. Madar, H. J. Maddocks, W. F. Mader, A. Madsen, J. Maeda, S. Maeland, T. Maeno, A. Maevskiy, E. Magradze, J. Mahlstedt, C. Maiani, C. Maidantchik, A. A. Maier, T. Maier, A. Maio, S. Majewski, Y. Makida, N. Makovec, B. Malaescu, Pa. Malecki, V. P. Maleev, F. Malek, U. Mallik, D. Malon, C. Malone, C. Malone, S. Maltezos, S. Malyukov, J. Mamuzic, G. Mancini, L. Mandelli, I. Mandić, J. Maneira, L. Manhaes de Andrade Filho, J. Manjarres Ramos, A. Mann, A. Manousos, B. Mansoulie, J. D. Mansour, R. Mantifel, M. Mantoani, S. Manzoni, L. Mapelli, G. Marceca, L. March, G. Marchiori, M. Marcisovsky, M. Marjanovic, D. E. Marley, F. Marroquim, S. P. Marsden, Z. Marshall, S. Marti-Garcia, B. Martin, T. A. Martin, V. J. Martin, B. Martin dit Latour, M. Martinez, V. I. Martinez Outschoorn, S. Martin-Haugh, V. S. Martoiu, A. C. Martyniuk, M. Marx, A. Marzin, L. Masetti, T. Mashimo, R. Mashinistov, J. Masik, A. L. Maslennikov, I. Massa, L. Massa, P. Mastrandrea, A. Mastroberardino, T. Masubuchi, P. Mättig, J. Mattmann, J. Maurer, S. J. Maxfield, D. A. Maximov, R. Mazini, S. M. Mazza, N. C. Mc Fadden, G. Mc Goldrick, S. P. Mc Kee, A. McCarn, R. L. McCarthy, T. G. McCarthy, L. I. McClymont, E. F. McDonald, J. A. Mcfayden, G. Mchedlidze, S. J. McMahon, R. A. McPherson, M. Medinnis, S. Meehan, S. Mehlhase, A. Mehta, K. Meier, C. Meineck, B. Meirose, D. Melini, B. R. Mellado Garcia, M. Melo, F. Meloni, A. Mengarelli, S. Menke, E. Meoni, S. Mergelmeyer, P. Mermod, L. Merola, C. Meroni, F. S. Merritt, A. Messina, J. Metcalfe, A. S. Mete, C. Meyer, C. Meyer, J-P. Meyer, J. Meyer, H. Meyer Zu Theenhausen, F. Miano, R. P. Middleton, S. Miglioranzi, L. Mijović, G. Mikenberg, M. Mikestikova, M. Mikuž, M. Milesi, A. Milic, D. W. Miller, C. Mills, A. Milov, D. A. Milstead, A. A. Minaenko, Y. Minami, I. A. Minashvili, A. I. Mincer, B. Mindur, M. Mineev, Y. Minegishi, Y. Ming, L. M. Mir, K. P. Mistry, T. Mitani, J. Mitrevski, V. A. Mitsou, A. Miucci, P. S. Miyagawa, J. U. Mjörnmark, M. Mlynarikova, T. Moa, K. Mochizuki, S. Mohapatra, S. Molander, R. Moles-Valls, R. Monden, M. C. Mondragon, K. Mönig, J. Monk, E. Monnier, A. Montalbano, J. Montejo Berlingen, F. Monticelli, S. Monzani, R. W. Moore, N. Morange, D. Moreno, M. Moreno Llácer, P. Morettini, S. Morgenstern, D. Mori, T. Mori, M. Morii, M. Morinaga, V. Morisbak, S. Moritz, A. K. Morley, G. Mornacchi, J. D. Morris, S. S. Mortensen, L. Morvaj, M. Mosidze, J. Moss, K. Motohashi, R. Mount, E. Mountricha, E. J. W. Moyse, S. Muanza, R. D. Mudd, F. Mueller, J. Mueller, R. S. P. Mueller, T. Mueller, D. Muenstermann, P. Mullen, G. A. Mullier, F. J. Munoz Sanchez, J. A. Murillo Quijada, W. J. Murray, H. Musheghyan, M. Muškinja, A. G. Myagkov, M. Myska, B. P. Nachman, O. Nackenhorst, K. Nagai, R. Nagai, K. Nagano, Y. Nagasaka, K. Nagata, M. Nagel, E. Nagy, A. M. Nairz, Y. Nakahama, K. Nakamura, T. Nakamura, I. Nakano, H. Namasivayam, R. F. Naranjo Garcia, R. Narayan, D. I. Narrias Villar, I. Naryshkin, T. Naumann, G. Navarro, R. Nayyar, H. A. Neal, P. Yu. Nechaeva, T. J. Neep, A. Negri, M. Negrini, S. Nektarijevic, C. Nellist, A. Nelson, S. Nemecek, P. Nemethy, A. A. Nepomuceno, M. Nessi, M. S. Neubauer, M. Neumann, R. M. Neves, P. Nevski, P. R. Newman, D. H. Nguyen, T. Nguyen Manh, R. B. Nickerson, R. Nicolaidou, J. Nielsen, A. Nikiforov, V. Nikolaenko, I. Nikolic-Audit, K. Nikolopoulos, J. K. Nilsen, P. Nilsson, Y. Ninomiya, A. Nisati, R. Nisius, T. Nobe, M. Nomachi, I. Nomidis, T. Nooney, S. Norberg, M. Nordberg, N. Norjoharuddeen, O. Novgorodova, S. Nowak, M. Nozaki, L. Nozka, K. Ntekas, E. Nurse, F. Nuti, F. O’grady, D. C. O’Neil, A. A. O’Rourke, V. O’Shea, F. G. Oakham, H. Oberlack, T. Obermann, J. Ocariz, A. Ochi, I. Ochoa, J. P. Ochoa-Ricoux, S. Oda, S. Odaka, H. Ogren, A. Oh, S. H. Oh, C. C. Ohm, H. Ohman, H. Oide, H. Okawa, Y. Okumura, T. Okuyama, A. Olariu, L. F. Oleiro Seabra, S. A. Olivares Pino, D. Oliveira Damazio, A. Olszewski, J. Olszowska, A. Onofre, K. Onogi, P. U. E. Onyisi, M. J. Oreglia, Y. Oren, D. Orestano, N. Orlando, R. S. Orr, B. Osculati, R. Ospanov, G. Otero y Garzon, H. Otono, M. Ouchrif, F. Ould-Saada, A. Ouraou, K. P. Oussoren, Q. Ouyang, M. Owen, R. E. Owen, V. E. Ozcan, N. Ozturk, K. Pachal, A. Pacheco Pages, L. Pacheco Rodriguez, C. Padilla Aranda, M. Pagáčová, S. Pagan Griso, M. Paganini, F. Paige, P. Pais, K. Pajchel, G. Palacino, S. Palazzo, S. Palestini, M. Palka, D. Pallin, E. St. Panagiotopoulou, C. E. Pandini, J. G. Panduro Vazquez, P. Pani, S. Panitkin, D. Pantea, L. Paolozzi, Th. D. Papadopoulou, K. Papageorgiou, A. Paramonov, D. Paredes Hernandez, A. J. Parker, M. A. Parker, K. A. Parker, F. Parodi, J. A. Parsons, U. Parzefall, V. R. Pascuzzi, E. Pasqualucci, S. Passaggio, Fr. Pastore, G. Pásztor, S. Pataraia, J. R. Pater, T. Pauly, J. Pearce, B. Pearson, L. E. Pedersen, M. Pedersen, S. Pedraza Lopez, R. Pedro, S. V. Peleganchuk, O. Penc, C. Peng, H. Peng, J. Penwell, B. S. Peralva, M. M. Perego, D. V. Perepelitsa, E. Perez Codina, L. Perini, H. Pernegger, S. Perrella, R. Peschke, V. D. Peshekhonov, K. Peters, R. F. Y. Peters, B. A. Petersen, T. C. Petersen, E. Petit, A. Petridis, C. Petridou, P. Petroff, E. Petrolo, M. Petrov, F. Petrucci, N. E. Pettersson, A. Peyaud, R. Pezoa, P. W. Phillips, G. Piacquadio, E. Pianori, A. Picazio, E. Piccaro, M. Piccinini, M. A. Pickering, R. Piegaia, J. E. Pilcher, A. D. Pilkington, A. W. J. Pin, M. Pinamonti, J. L. Pinfold, A. Pingel, S. Pires, H. Pirumov, M. Pitt, L. Plazak, M. -A. Pleier, V. Pleskot, E. Plotnikova, P. Plucinski, D. Pluth, R. Poettgen, L. Poggioli, D. Pohl, G. Polesello, A. Poley, A. Policicchio, R. Polifka, A. Polini, C. S. Pollard, V. Polychronakos, K. Pommès, L. Pontecorvo, B. G. Pope, G. A. Popeneciu, A. Poppleton, S. Pospisil, K. Potamianos, I. N. Potrap, C. J. Potter, C. T. Potter, G. Poulard, J. Poveda, V. Pozdnyakov, M. E. Pozo Astigarraga, P. Pralavorio, A. Pranko, S. Prell, D. Price, L. E. Price, M. Primavera, S. Prince, K. Prokofiev, F. Prokoshin, S. Protopopescu, J. Proudfoot, M. Przybycien, D. Puddu, M. Purohit, P. Puzo, J. Qian, G. Qin, Y. Qin, A. Quadt, W. B. Quayle, M. Queitsch-Maitland, D. Quilty, S. Raddum, V. Radeka, V. Radescu, S. K. Radhakrishnan, P. Radloff, P. Rados, F. Ragusa, G. Rahal, J. A. Raine, S. Rajagopalan, M. Rammensee, C. Rangel-Smith, M. G. Ratti, F. Rauscher, S. Rave, T. Ravenscroft, I. Ravinovich, M. Raymond, A. L. Read, N. P. Readioff, M. Reale, D. M. Rebuzzi, A. Redelbach, G. Redlinger, R. Reece, R. G. Reed, K. Reeves, L. Rehnisch, J. Reichert, A. Reiss, C. Rembser, H. Ren, M. Rescigno, S. Resconi, O. L. Rezanova, P. Reznicek, R. Rezvani, R. Richter, S. Richter, E. Richter-Was, O. Ricken, M. Ridel, P. Rieck, C. J. Riegel, J. Rieger, O. Rifki, M. Rijssenbeek, A. Rimoldi, M. Rimoldi, L. Rinaldi, B. Ristić, E. Ritsch, I. Riu, F. Rizatdinova, E. Rizvi, C. Rizzi, S. H. Robertson, A. Robichaud-Veronneau, D. Robinson, J. E. M. Robinson, A. Robson, C. Roda, Y. Rodina, A. Rodriguez Perez, D. Rodriguez Rodriguez, S. Roe, C. S. Rogan, O. Røhne, A. Romaniouk, M. Romano, S. M. Romano Saez, E. Romero Adam, N. Rompotis, M. Ronzani, L. Roos, E. Ros, S. Rosati, K. Rosbach, P. Rose, N. -A. Rosien, V. Rossetti, E. Rossi, L. P. Rossi, J. H. N. Rosten, R. Rosten, M. Rotaru, I. Roth, J. Rothberg, D. Rousseau, A. Rozanov, Y. Rozen, X. Ruan, F. Rubbo, M. S. Rudolph, F. Rühr, A. Ruiz-Martinez, Z. Rurikova, N. A. Rusakovich, A. Ruschke, H. L. Russell, J. P. Rutherfoord, N. Ruthmann, Y. F. Ryabov, M. Rybar, G. Rybkin, S. Ryu, A. Ryzhov, G. F. Rzehorz, A. F. Saavedra, G. Sabato, S. Sacerdoti, H. F-W. Sadrozinski, R. Sadykov, F. Safai Tehrani, P. Saha, M. Sahinsoy, M. Saimpert, T. Saito, H. Sakamoto, Y. Sakurai, G. Salamanna, A. Salamon, J. E. Salazar Loyola, D. Salek, P. H. Sales De Bruin, D. Salihagic, A. Salnikov, J. Salt, D. Salvatore, F. Salvatore, A. Salvucci, A. Salzburger, D. Sammel, D. Sampsonidis, A. Sanchez, J. Sánchez, V. Sanchez Martinez, H. Sandaker, R. L. Sandbach, H. G. Sander, M. Sandhoff, C. Sandoval, D. P. C. Sankey, M. Sannino, A. Sansoni, C. Santoni, R. Santonico, H. Santos, I. Santoyo Castillo, K. Sapp, A. Sapronov, J. G. Saraiva, B. Sarrazin, O. Sasaki, K. Sato, E. Sauvan, G. Savage, P. Savard, N. Savic, C. Sawyer, L. Sawyer, J. Saxon, C. Sbarra, A. Sbrizzi, T. Scanlon, D. A. Scannicchio, M. Scarcella, V. Scarfone, J. Schaarschmidt, P. Schacht, B. M. Schachtner, D. Schaefer, L. Schaefer, R. Schaefer, J. Schaeffer, S. Schaepe, S. Schaetzel, U. Schäfer, A. C. Schaffer, D. Schaile, R. D. Schamberger, V. Scharf, V. A. Schegelsky, D. Scheirich, M. Schernau, C. Schiavi, S. Schier, C. Schillo, M. Schioppa, S. Schlenker, K. R. Schmidt-Sommerfeld, K. Schmieden, C. Schmitt, S. Schmitt, S. Schmitz, B. Schneider, U. Schnoor, L. Schoeffel, A. Schoening, B. D. Schoenrock, E. Schopf, M. Schott, J. F. P. Schouwenberg, J. Schovancova, S. Schramm, M. Schreyer, N. Schuh, A. Schulte, M. J. Schultens, H. -C. Schultz-Coulon, H. Schulz, M. Schumacher, B. A. Schumm, Ph. Schune, A. Schwartzman, T. A. Schwarz, H. Schweiger, Ph. Schwemling, R. Schwienhorst, J. Schwindling, T. Schwindt, G. Sciolla, F. Scuri, F. Scutti, J. Searcy, P. Seema, S. C. Seidel, A. Seiden, F. Seifert, J. M. Seixas, G. Sekhniaidze, K. Sekhon, S. J. Sekula, D. M. Seliverstov, N. Semprini-Cesari, C. Serfon, L. Serin, L. Serkin, M. Sessa, R. Seuster, H. Severini, T. Sfiligoj, F. Sforza, A. Sfyrla, E. Shabalina, N. W. Shaikh, L. Y. Shan, R. Shang, J. T. Shank, M. Shapiro, P. B. Shatalov, K. Shaw, S. M. Shaw, A. Shcherbakova, C. Y. Shehu, P. Sherwood, L. Shi, S. Shimizu, C. O. Shimmin, M. Shimojima, S. Shirabe, M. Shiyakova, A. Shmeleva, D. Shoaleh Saadi, M. J. Shochet, S. Shojaii, D. R. Shope, S. Shrestha, E. Shulga, M. A. Shupe, P. Sicho, A. M. Sickles, P. E. Sidebo, O. Sidiropoulou, D. Sidorov, A. Sidoti, F. Siegert, Dj. Sijacki, J. Silva, S. B. Silverstein, V. Simak, Lj. Simic, S. Simion, E. Simioni, B. Simmons, D. Simon, M. Simon, P. Sinervo, N. B. Sinev, M. Sioli, G. Siragusa, S. Yu. Sivoklokov, J. Sjölin, M. B. Skinner, H. P. Skottowe, P. Skubic, M. Slater, T. Slavicek, M. Slawinska, K. Sliwa, R. Slovak, V. Smakhtin, B. H. Smart, L. Smestad, J. Smiesko, S. Yu. Smirnov, Y. Smirnov, L. N. Smirnova, O. Smirnova, M. N. K. Smith, R. W. Smith, M. Smizanska, K. Smolek, A. A. Snesarev, I. M. Snyder, S. Snyder, R. Sobie, F. Socher, A. Soffer, D. A. Soh, G. Sokhrannyi, C. A. Solans Sanchez, M. Solar, E. Yu. Soldatov, U. Soldevila, A. A. Solodkov, A. Soloshenko, O. V. Solovyanov, V. Solovyev, P. Sommer, H. Son, H. Y. Song, A. Sood, A. Sopczak, V. Sopko, V. Sorin, D. Sosa, C. L. Sotiropoulou, R. Soualah, A. M. Soukharev, D. South, B. C. Sowden, S. Spagnolo, M. Spalla, M. Spangenberg, F. Spanò, D. Sperlich, F. Spettel, R. Spighi, G. Spigo, L. A. Spiller, M. Spousta, R. D. St. Denis, A. Stabile, R. Stamen, S. Stamm, E. Stanecka, R. W. Stanek, C. Stanescu, M. Stanescu-Bellu, M. M. Stanitzki, S. Stapnes, E. A. Starchenko, G. H. Stark, J. Stark, P. Staroba, P. Starovoitov, S. Stärz, R. Staszewski, P. Steinberg, B. Stelzer, H. J. Stelzer, O. Stelzer-Chilton, H. Stenzel, G. A. Stewart, J. A. Stillings, M. C. Stockton, M. Stoebe, G. Stoicea, P. Stolte, S. Stonjek, A. R. Stradling, A. Straessner, M. E. Stramaglia, J. Strandberg, S. Strandberg, A. Strandlie, M. Strauss, P. Strizenec, R. Ströhmer, D. M. Strom, R. Stroynowski, A. Strubig, S. A. Stucci, B. Stugu, N. A. Styles, D. Su, J. Su, S. Suchek, Y. Sugaya, M. Suk, V. V. Sulin, S. Sultansoy, T. Sumida, S. Sun, X. Sun, J. E. Sundermann, K. Suruliz, G. Susinno, M. R. Sutton, S. Suzuki, M. Svatos, M. Swiatlowski, I. Sykora, T. Sykora, D. Ta, C. Taccini, K. Tackmann, J. Taenzer, A. Taffard, R. Tafirout, N. Taiblum, H. Takai, R. Takashima, T. Takeshita, Y. Takubo, M. Talby, A. A. Talyshev, K. G. Tan, J. Tanaka, M. Tanaka, R. Tanaka, S. Tanaka, R. Tanioka, B. B. Tannenwald, S. Tapia Araya, S. Tapprogge, S. Tarem, G. F. Tartarelli, P. Tas, M. Tasevsky, T. Tashiro, E. Tassi, A. Tavares Delgado, Y. Tayalati, A. C. Taylor, G. N. Taylor, P. T. E. Taylor, W. Taylor, F. A. Teischinger, P. Teixeira-Dias, K. K. Temming, D. Temple, H. Ten Kate, P. K. Teng, J. J. Teoh, F. Tepel, S. Terada, K. Terashi, J. Terron, S. Terzo, M. Testa, R. J. Teuscher, T. Theveneaux-Pelzer, J. P. Thomas, J. Thomas-Wilsker, E. N. Thompson, P. D. Thompson, A. S. Thompson, L. A. Thomsen, E. Thomson, M. Thomson, M. J. Tibbetts, R. E. Ticse Torres, V. O. Tikhomirov, Yu. A. Tikhonov, S. Timoshenko, P. Tipton, S. Tisserant, K. Todome, T. Todorov, S. Todorova-Nova, J. Tojo, S. Tokár, K. Tokushuku, E. Tolley, L. Tomlinson, M. Tomoto, L. Tompkins, K. Toms, B. Tong, P. Tornambe, E. Torrence, H. Torres, E. Torró Pastor, J. Toth, F. Touchard, D. R. Tovey, T. Trefzger, A. Tricoli, I. M. Trigger, S. Trincaz-Duvoid, M. F. Tripiana, W. Trischuk, B. Trocmé, A. Trofymov, C. Troncon, M. Trottier-McDonald, M. Trovatelli, L. Truong, M. Trzebinski, A. Trzupek, J. C-L. Tseng, P. V. Tsiareshka, G. Tsipolitis, N. Tsirintanis, S. Tsiskaridze, V. Tsiskaridze, E. G. Tskhadadze, K. M. Tsui, I. I. Tsukerman, V. Tsulaia, S. Tsuno, D. Tsybychev, Y. Tu, A. Tudorache, V. Tudorache, A. N. Tuna, S. A. Tupputi, S. Turchikhin, D. Turecek, D. Turgeman, R. Turra, P. M. Tuts, M. Tyndel, G. Ucchielli, I. Ueda, M. Ughetto, F. Ukegawa, G. Unal, A. Undrus, G. Unel, F. C. Ungaro, Y. Unno, C. Unverdorben, J. Urban, P. Urquijo, P. Urrejola, G. Usai, L. Vacavant, V. Vacek, B. Vachon, C. Valderanis, E. Valdes Santurio, N. Valencic, S. Valentinetti, A. Valero, L. Valery, S. Valkar, J. A. Valls Ferrer, W. Van Den Wollenberg, P. C. Van Der Deijl, H. van der Graaf, N. van Eldik, P. van Gemmeren, J. Van Nieuwkoop, I. van Vulpen, M. C. van Woerden, M. Vanadia, W. Vandelli, R. Vanguri, A. Vaniachine, P. Vankov, G. Vardanyan, R. Vari, E. W. Varnes, T. Varol, D. Varouchas, A. Vartapetian, K. E. Varvell, J. G. Vasquez, G. A. Vasquez, F. Vazeille, T. Vazquez Schroeder, J. Veatch, V. Veeraraghavan, L. M. Veloce, F. Veloso, S. Veneziano, A. Ventura, M. Venturi, N. Venturi, A. Venturini, V. Vercesi, M. Verducci, W. Verkerke, J. C. Vermeulen, A. Vest, M. C. Vetterli, O. Viazlo, I. Vichou, T. Vickey, O. E. Vickey Boeriu, G. H. A. Viehhauser, S. Viel, L. Vigani, M. Villa, M. Villaplana Perez, E. Vilucchi, M. G. Vincter, V. B. Vinogradov, C. Vittori, I. Vivarelli, S. Vlachos, M. Vlasak, M. Vogel, P. Vokac, G. Volpi, M. Volpi, H. von der Schmitt, E. von Toerne, V. Vorobel, K. Vorobev, M. Vos, R. Voss, J. H. Vossebeld, N. Vranjes, M. Vranjes Milosavljevic, V. Vrba, M. Vreeswijk, R. Vuillermet, I. Vukotic, Z. Vykydal, P. Wagner, W. Wagner, H. Wahlberg, S. Wahrmund, J. Wakabayashi, J. Walder, R. Walker, W. Walkowiak, V. Wallangen, C. Wang, C. Wang, F. Wang, H. Wang, H. Wang, J. Wang, J. Wang, K. Wang, R. Wang, S. M. Wang, T. Wang, T. Wang, W. Wang, X. Wang, C. Wanotayaroj, A. Warburton, C. P. Ward, D. R. Wardrope, A. Washbrook, P. M. Watkins, A. T. Watson, M. F. Watson, G. Watts, S. Watts, B. M. Waugh, S. Webb, M. S. Weber, S. W. Weber, S. A. Weber, J. S. Webster, A. R. Weidberg, B. Weinert, J. Weingarten, C. Weiser, H. Weits, P. S. Wells, T. Wenaus, T. Wengler, S. Wenig, N. Wermes, M. Werner, M. D. Werner, P. Werner, M. Wessels, J. Wetter, K. Whalen, N. L. Whallon, A. M. Wharton, A. White, M. J. White, R. White, D. Whiteson, F. J. Wickens, W. Wiedenmann, M. Wielers, C. Wiglesworth, L. A. M. Wiik-Fuchs, A. Wildauer, F. Wilk, H. G. Wilkens, H. H. Williams, S. Williams, C. Willis, S. Willocq, J. A. Wilson, I. Wingerter-Seez, F. Winklmeier, O. J. Winston, B. T. Winter, M. Wittgen, J. Wittkowski, T. M. H. Wolf, M. W. Wolter, H. Wolters, S. D. Worm, B. K. Wosiek, J. Wotschack, M. J. Woudstra, K. W. Wozniak, M. Wu, M. Wu, S. L. Wu, X. Wu, Y. Wu, T. R. Wyatt, B. M. Wynne, S. Xella, D. Xu, L. Xu, B. Yabsley, S. Yacoob, D. Yamaguchi, Y. Yamaguchi, A. Yamamoto, S. Yamamoto, T. Yamanaka, K. Yamauchi, Y. Yamazaki, Z. Yan, H. Yang, H. Yang, Y. Yang, Z. Yang, W-M. Yao, Y. C. Yap, Y. Yasu, E. Yatsenko, K. H. Yau Wong, J. Ye, S. Ye, I. Yeletskikh, A. L. Yen, E. Yildirim, K. Yorita, R. Yoshida, K. Yoshihara, C. Young, C. J. S. Young, S. Youssef, D. R. Yu, J. Yu, J. M. Yu, J. Yu, L. Yuan, S. P. Y. Yuen, I. Yusuff, B. Zabinski, R. Zaidan, A. M. Zaitsev, N. Zakharchuk, J. Zalieckas, A. Zaman, S. Zambito, L. Zanello, D. Zanzi, C. Zeitnitz, M. Zeman, A. Zemla, J. C. Zeng, Q. Zeng, K. Zengel, O. Zenin, T. Ženiš, D. Zerwas, D. Zhang, F. Zhang, G. Zhang, H. Zhang, J. Zhang, L. Zhang, R. Zhang, R. Zhang, X. Zhang, Z. Zhang, X. Zhao, Y. Zhao, Z. Zhao, A. Zhemchugov, J. Zhong, B. Zhou, C. Zhou, L. Zhou, L. Zhou, M. Zhou, N. Zhou, C. G. Zhu, H. Zhu, J. Zhu, Y. Zhu, X. Zhuang, K. Zhukov, A. Zibell, D. Zieminska, N. I. Zimine, C. Zimmermann, S. Zimmermann, Z. Zinonos, M. Zinser, M. Ziolkowski, L. Živković, G. Zobernig, A. Zoccoli, M. zur Nedden, L. Zwalinski, ATLAS Collaboration

**Affiliations:** 1 Department of Physics, University of Adelaide, Adelaide, Australia; 2 Physics Department, SUNY Albany, Albany, NY USA; 3 Department of Physics, University of Alberta, Edmonton, AB Canada; 4 Department of Physics, Ankara University, Ankara, Turkey; 5 Istanbul Aydin University, Istanbul, Turkey; 6 Division of Physics, TOBB University of Economics and Technology, Ankara, Turkey; 7 LAPP, CNRS/IN2P3 and Université Savoie Mont Blanc, Annecy-le-Vieux, France; 8 High Energy Physics Division, Argonne National Laboratory, Argonne, IL USA; 9 Department of Physics, University of Arizona, Tucson, AZ USA; 10 Department of Physics, The University of Texas at Arlington, Arlington, TX USA; 11 Physics Department, University of Athens, Athens, Greece; 12 Physics Department, National Technical University of Athens, Zografou, Greece; 13 Department of Physics, The University of Texas at Austin, Austin, TX USA; 14 Institute of Physics, Azerbaijan Academy of Sciences, Baku, Azerbaijan; 15 Institut de Física d’Altes Energies (IFAE), The Barcelona Institute of Science and Technology, Barcelona, Spain; 16 Institute of Physics, University of Belgrade, Belgrade, Serbia; 17 Department for Physics and Technology, University of Bergen, Bergen, Norway; 18 Physics Division, Lawrence Berkeley National Laboratory and University of California, Berkeley, CA USA; 19 Department of Physics, Humboldt University, Berlin, Germany; 20 Albert Einstein Center for Fundamental Physics and Laboratory for High Energy Physics, University of Bern, Bern, Switzerland; 21 School of Physics and Astronomy, University of Birmingham, Birmingham, UK; 22 Department of Physics, Bogazici University, Istanbul, Turkey; 23 Department of Physics Engineering, Gaziantep University, Gaziantep, Turkey; 24 Faculty of Engineering and Natural Sciences, Istanbul Bilgi University, Istanbul, Turkey; 25 Faculty of Engineering and Natural Sciences, Bahcesehir University, Istanbul, Turkey; 26 Centro de Investigaciones, Universidad Antonio Narino, Bogota, Colombia; 27 INFN Sezione di Bologna, Bologna, Italy; 28 Dipartimento di Fisica e Astronomia, Università di Bologna, Bologna, Italy; 29 Physikalisches Institut, University of Bonn, Bonn, Germany; 30 Department of Physics, Boston University, Boston, MA USA; 31 Department of Physics, Brandeis University, Waltham, MA USA; 32 Universidade Federal do Rio De Janeiro COPPE/EE/IF, Rio de Janeiro, Brazil; 33 Electrical Circuits Department, Federal University of Juiz de Fora (UFJF), Juiz de Fora, Brazil; 34 Federal University of Sao Joao del Rei (UFSJ), Sao Joao del Rei, Brazil; 35 Instituto de Fisica, Universidade de Sao Paulo, São Paulo, Brazil; 36 Physics Department, Brookhaven National Laboratory, Upton, NY USA; 37 Transilvania University of Brasov, Brasov, Romania; 38 National Institute of Physics and Nuclear Engineering, Bucharest, Romania; 39 Physics Department, National Institute for Research and Development of Isotopic and Molecular Technologies, Cluj Napoca, Romania; 40 University Politehnica Bucharest, Bucharest, Romania; 41 West University in Timisoara, Timisoara, Romania; 42 Departamento de Física, Universidad de Buenos Aires, Buenos Aires, Argentina; 43 Cavendish Laboratory, University of Cambridge, Cambridge, UK; 44 Department of Physics, Carleton University, Ottawa, ON Canada; 45 CERN, Geneva, Switzerland; 46 Enrico Fermi Institute, University of Chicago, Chicago, IL USA; 47 Departamento de Física, Pontificia Universidad Católica de Chile, Santiago, Chile; 48 Departamento de Física, Universidad Técnica Federico Santa María, Valparaiso, Chile; 49 Institute of High Energy Physics, Chinese Academy of Sciences, Beijing, China; 50 Department of Physics, Nanjing University, Jiangsu, China; 51 Physics Department, Tsinghua University, Beijing, 100084 China; 52 Laboratoire de Physique Corpusculaire, Clermont Université and Université Blaise Pascal and CNRS/IN2P3, Clermont-Ferrand, France; 53 Nevis Laboratory, Columbia University, Irvington, NY USA; 54 Niels Bohr Institute, University of Copenhagen, Kobenhavn, Denmark; 55 Laboratori Nazionali di Frascati, INFN Gruppo Collegato di Cosenza, Frascati, Italy; 56 Dipartimento di Fisica, Università della Calabria, Rende, Italy; 57 Faculty of Physics and Applied Computer Science, AGH University of Science and Technology, Kraków, Poland; 58 Marian Smoluchowski Institute of Physics, Jagiellonian University, Kraków, Poland; 59 Institute of Nuclear Physics, Polish Academy of Sciences, Kraków, Poland; 60 Physics Department, Southern Methodist University, Dallas, TX USA; 61 Physics Department, University of Texas at Dallas, Richardson, TX USA; 62 DESY, Hamburg and Zeuthen, Germany; 63 Lehrstuhl für Experimentelle Physik IV, Technische Universität Dortmund, Dortmund, Germany; 64 Institut für Kern- und Teilchenphysik, Technische Universität Dresden, Dresden, Germany; 65 Department of Physics, Duke University, Durham, NC USA; 66 SUPA-School of Physics and Astronomy, University of Edinburgh, Edinburgh, UK; 67 INFN Laboratori Nazionali di Frascati, Frascati, Italy; 68 Fakultät für Mathematik und Physik, Albert-Ludwigs-Universität, Freiburg, Germany; 69 Section de Physique, Université de Genève, Geneva, Switzerland; 70 INFN Sezione di Genova, Genoa, Italy; 71 Dipartimento di Fisica, Università di Genova, Genoa, Italy; 72 E. Andronikashvili Institute of Physics, Iv. Javakhishvili Tbilisi State University, Tbilisi, Georgia; 73 High Energy Physics Institute, Tbilisi State University, Tbilisi, Georgia; 74 II Physikalisches Institut, Justus-Liebig-Universität Giessen, Giessen, Germany; 75 SUPA-School of Physics and Astronomy, University of Glasgow, Glasgow, UK; 76 II Physikalisches Institut, Georg-August-Universität, Göttingen, Germany; 77 Laboratoire de Physique Subatomique et de Cosmologie, Université Grenoble-Alpes, CNRS/IN2P3, Grenoble, France; 78 Laboratory for Particle Physics and Cosmology, Harvard University, Cambridge, MA USA; 79 Department of Modern Physics, University of Science and Technology of China, Anhui, China; 80 Kirchhoff-Institut für Physik, Ruprecht-Karls-Universität Heidelberg, Heidelberg, Germany; 81 Physikalisches Institut, Ruprecht-Karls-Universität Heidelberg, Heidelberg, Germany; 82 ZITI Institut für technische Informatik, Ruprecht-Karls-Universität Heidelberg, Mannheim, Germany; 83 Faculty of Applied Information Science, Hiroshima Institute of Technology, Hiroshima, Japan; 84 Department of Physics, The Chinese University of Hong Kong, Shatin, N.T., Hong Kong, China; 85 Department of Physics, The University of Hong Kong, Hong Kong, China; 86 Department of Physics, The Hong Kong University of Science and Technology, Clear Water Bay, Kowloon, Hong Kong China; 87 Department of Physics, Indiana University, Bloomington, IN USA; 88 Institut für Astro- und Teilchenphysik, Leopold-Franzens-Universität, Innsbruck, Austria; 89 University of Iowa, Iowa City, IA USA; 90 Department of Physics and Astronomy, Iowa State University, Ames, IA USA; 91 Joint Institute for Nuclear Research, JINR Dubna, Dubna, Russia; 92 KEK, High Energy Accelerator Research Organization, Tsukuba, Japan; 93 Graduate School of Science, Kobe University, Kobe, Japan; 94 Faculty of Science, Kyoto University, Kyoto, Japan; 95 Kyoto University of Education, Kyoto, Japan; 96 Department of Physics, Kyushu University, Fukuoka, Japan; 97 Instituto de Física La Plata, Universidad Nacional de La Plata and CONICET, La Plata, Argentina; 98 Physics Department, Lancaster University, Lancaster, UK; 99 INFN Sezione di Lecce, Lecce, Italy; 100 Dipartimento di Matematica e Fisica, Università del Salento, Lecce, Italy; 101 Oliver Lodge Laboratory, University of Liverpool, Liverpool, UK; 102 Department of Physics, Jožef Stefan Institute and University of Ljubljana, Ljubljana, Slovenia; 103 School of Physics and Astronomy, Queen Mary University of London, London, UK; 104 Department of Physics, Royal Holloway University of London, Surrey, UK; 105 Department of Physics and Astronomy, University College London, London, UK; 106 Louisiana Tech University, Ruston, LA USA; 107 Laboratoire de Physique Nucléaire et de Hautes Energies, UPMC and Université Paris-Diderot and CNRS/IN2P3, Paris, France; 108 Fysiska Institutionen, Lunds Universitet, Lund, Sweden; 109 Departamento de Fisica Teorica C-15, Universidad Autonoma de Madrid, Madrid, Spain; 110 Institut für Physik, Universität Mainz, Mainz, Germany; 111 School of Physics and Astronomy, University of Manchester, Manchester, UK; 112 CPPM, Aix-Marseille Université and CNRS/IN2P3, Marseille, France; 113 Department of Physics, University of Massachusetts, Amherst, MA USA; 114 Department of Physics, McGill University, Montreal, QC Canada; 115 School of Physics, University of Melbourne, Melbourne, VIC Australia; 116 Department of Physics, The University of Michigan, Ann Arbor, MI USA; 117 Department of Physics and Astronomy, Michigan State University, East Lansing, MI USA; 118 INFN Sezione di Milano, Milan, Italy; 119 Dipartimento di Fisica, Università di Milano, Milan, Italy; 120 B.I. Stepanov Institute of Physics, National Academy of Sciences of Belarus, Minsk, Republic of Belarus; 121 National Scientific and Educational Centre for Particle and High Energy Physics, Minsk, Republic of Belarus; 122 Group of Particle Physics, University of Montreal, Montreal, QC Canada; 123 P.N. Lebedev Physical Institute of the Russian, Academy of Sciences, Moscow, Russia; 124 Institute for Theoretical and Experimental Physics (ITEP), Moscow, Russia; 125 National Research Nuclear University MEPhI, Moscow, Russia; 126 D.V. Skobeltsyn Institute of Nuclear Physics, M.V. Lomonosov Moscow State University, Moscow, Russia; 127 Fakultät für Physik, Ludwig-Maximilians-Universität München, Munich, Germany; 128 Max-Planck-Institut für Physik (Werner-Heisenberg-Institut), Munich, Germany; 129 Nagasaki Institute of Applied Science, Nagasaki, Japan; 130 Graduate School of Science and Kobayashi-Maskawa Institute, Nagoya University, Nagoya, Japan; 131 INFN Sezione di Napoli, Naples, Italy; 132 Dipartimento di Fisica, Università di Napoli, Naples, Italy; 133 Department of Physics and Astronomy, University of New Mexico, Albuquerque, NM USA; 134 Institute for Mathematics, Astrophysics and Particle Physics, Radboud University Nijmegen/Nikhef, Nijmegen, The Netherlands; 135 Nikhef National Institute for Subatomic Physics and University of Amsterdam, Amsterdam, The Netherlands; 136 Department of Physics, Northern Illinois University, DeKalb, IL USA; 137 Budker Institute of Nuclear Physics, SB RAS, Novosibirsk, Russia; 138 Department of Physics, New York University, New York, NY USA; 139 Ohio State University, Columbus, OH USA; 140 Faculty of Science, Okayama University, Okayama, Japan; 141 Homer L. Dodge Department of Physics and Astronomy, University of Oklahoma, Norman, OK USA; 142 Department of Physics, Oklahoma State University, Stillwater, OK USA; 143 Palacký University, RCPTM, Olomouc, Czech Republic; 144 Center for High Energy Physics, University of Oregon, Eugene, OR USA; 145 LAL, University of Paris-Sud, CNRS/IN2P3, Université Paris-Saclay, Orsay, France; 146 Graduate School of Science, Osaka University, Osaka, Japan; 147 Department of Physics, University of Oslo, Oslo, Norway; 148 Department of Physics, Oxford University, Oxford, UK; 149 INFN Sezione di Pavia, Pavia, Italy; 150 Dipartimento di Fisica, Università di Pavia, Pavia, Italy; 151 Department of Physics, University of Pennsylvania, Philadelphia, PA USA; 152 National Research Centre “Kurchatov Institute” B.P.Konstantinov Petersburg Nuclear Physics Institute, St. Petersburg, Russia; 153 INFN Sezione di Pisa, Pisa, Italy; 154 Dipartimento di Fisica E. Fermi, Università di Pisa, Pisa, Italy; 155 Department of Physics and Astronomy, University of Pittsburgh, Pittsburgh, PA USA; 156 Laboratório de Instrumentação e Física Experimental de Partículas-LIP, Lisbon, Portugal; 157 Faculdade de Ciências, Universidade de Lisboa, Lisbon, Portugal; 158 Department of Physics, University of Coimbra, Coimbra, Portugal; 159 Centro de Física Nuclear da Universidade de Lisboa, Lisbon, Portugal; 160 Departamento de Fisica, Universidade do Minho, Braga, Portugal; 161 Departamento de Fisica Teorica y del Cosmos and CAFPE, Universidad de Granada, Granada, Spain; 162 Dep Fisica and CEFITEC of Faculdade de Ciencias e Tecnologia, Universidade Nova de Lisboa, Caparica, Portugal; 163 Institute of Physics, Academy of Sciences of the Czech Republic, Prague, Czech Republic; 164 Czech Technical University in Prague, Prague, Czech Republic; 165 Faculty of Mathematics and Physics, Charles University in Prague, Prague, Czech Republic; 166 State Research Center Institute for High Energy Physics (Protvino), NRC KI, Protvino, Russia; 167 Particle Physics Department, Rutherford Appleton Laboratory, Didcot, UK; 168 INFN Sezione di Roma, Rome, Italy; 169 Dipartimento di Fisica, Sapienza Università di Roma, Rome, Italy; 170 INFN Sezione di Roma Tor Vergata, Rome, Italy; 171 Dipartimento di Fisica, Università di Roma Tor Vergata, Rome, Italy; 172 INFN Sezione di Roma Tre, Rome, Italy; 173 Dipartimento di Matematica e Fisica, Università Roma Tre, Rome, Italy; 174 Faculté des Sciences Ain Chock, Réseau Universitaire de Physique des Hautes Energies-Université Hassan II, Casablanca, Morocco; 175 Centre National de l’Energie des Sciences Techniques Nucleaires, Rabat, Morocco; 176 Faculté des Sciences Semlalia, Université Cadi Ayyad, LPHEA-Marrakech, Marrakech, Morocco; 177 Faculté des Sciences, Université Mohamed Premier and LPTPM, Oujda, Morocco; 178 Faculté des Sciences, Université Mohammed V, Rabat, Morocco; 179 DSM/IRFU (Institut de Recherches sur les Lois Fondamentales de l’Univers), CEA Saclay (Commissariat à l’Energie Atomique et aux Energies Alternatives), Gif-sur-Yvette, France; 180 Santa Cruz Institute for Particle Physics, University of California Santa Cruz, Santa Cruz, CA USA; 181 Department of Physics, University of Washington, Seattle, WA USA; 182 School of Physics, Shandong University, Shandong, China; 183 Department of Physics and Astronomy, Shanghai Key Laboratory for Particle Physics and Cosmology, Shanghai Jiao Tong University (also affiliated with PKU-CHEP), Shanghai, China; 184 Department of Physics and Astronomy, University of Sheffield, Sheffield, UK; 185 Department of Physics, Shinshu University, Nagano, Japan; 186 Fachbereich Physik, Universität Siegen, Siegen, Germany; 187 Department of Physics, Simon Fraser University, Burnaby, BC Canada; 188 SLAC National Accelerator Laboratory, Stanford, CA USA; 189 Faculty of Mathematics, Physics and Informatics, Comenius University, Bratislava, Slovak Republic; 190 Department of Subnuclear Physics, Institute of Experimental Physics of the Slovak Academy of Sciences, Kosice, Slovak Republic; 191 Department of Physics, University of Cape Town, Cape Town, South Africa; 192 Department of Physics, University of Johannesburg, Johannesburg, South Africa; 193 School of Physics, University of the Witwatersrand, Johannesburg, South Africa; 194 Department of Physics, Stockholm University, Stockholm, Sweden; 195 The Oskar Klein Centre, Stockholm, Sweden; 196 Physics Department, Royal Institute of Technology, Stockholm, Sweden; 197 Departments of Physics and Astronomy and Chemistry, Stony Brook University, Stony Brook, NY USA; 198 Department of Physics and Astronomy, University of Sussex, Brighton, UK; 199 School of Physics, University of Sydney, Sydney, Australia; 200 Institute of Physics, Academia Sinica, Taipei, Taiwan; 201 Department of Physics, Technion: Israel Institute of Technology, Haifa, Israel; 202 Raymond and Beverly Sackler School of Physics and Astronomy, Tel Aviv University, Tel Aviv, Israel; 203 Department of Physics, Aristotle University of Thessaloniki, Thessaloníki, Greece; 204 International Center for Elementary Particle Physics and Department of Physics, The University of Tokyo, Tokyo, Japan; 205 Graduate School of Science and Technology, Tokyo Metropolitan University, Tokyo, Japan; 206 Department of Physics, Tokyo Institute of Technology, Tokyo, Japan; 207 Tomsk State University, Tomsk, Russia, Russia; 208 Department of Physics, University of Toronto, Toronto, ON Canada; 209 INFN-TIFPA, Povo, Italy; 210 University of Trento, Trento, Italy; 211 TRIUMF, Vancouver, BC Canada; 212 Department of Physics and Astronomy, York University, Toronto, ON Canada; 213 Faculty of Pure and Applied Sciences, and Center for Integrated Research in Fundamental Science and Engineering, University of Tsukuba, Tsukuba, Japan; 214 Department of Physics and Astronomy, Tufts University, Medford, MA USA; 215 Department of Physics and Astronomy, University of California Irvine, Irvine, CA USA; 216 INFN Gruppo Collegato di Udine, Sezione di Trieste, Udine, Italy; 217 ICTP, Trieste, Italy; 218 Dipartimento di Chimica Fisica e Ambiente, Università di Udine, Udine, Italy; 219 Department of Physics and Astronomy, University of Uppsala, Uppsala, Sweden; 220 Department of Physics, University of Illinois, Urbana, IL USA; 221 Instituto de Fisica Corpuscular (IFIC) and Departamento de Fisica Atomica, Molecular y Nuclear and Departamento de Ingeniería Electrónica and Instituto de Microelectrónica de Barcelona (IMB-CNM), University of Valencia and CSIC, Valencia, Spain; 222 Department of Physics, University of British Columbia, Vancouver, BC Canada; 223 Department of Physics and Astronomy, University of Victoria, Victoria, BC Canada; 224 Department of Physics, University of Warwick, Coventry, UK; 225 Waseda University, Tokyo, Japan; 226 Department of Particle Physics, The Weizmann Institute of Science, Rehovot, Israel; 227 Department of Physics, University of Wisconsin, Madison, WI USA; 228 Fakultät für Physik und Astronomie, Julius-Maximilians-Universität, Würzburg, Germany; 229 Fakultät für Mathematik und Naturwissenschaften, Fachgruppe Physik, Bergische Universität Wuppertal, Wuppertal, Germany; 230 Department of Physics, Yale University, New Haven, CT USA; 231 Yerevan Physics Institute, Yerevan, Armenia; 232 Centre de Calcul de l’Institut National de Physique Nucléaire et de Physique des Particules (IN2P3), Villeurbanne, France; 233 CERN, 1211 Geneva 23, Switzerland

## Abstract

A search is performed for a heavy particle decaying into different flavour dilepton pairs ($e\mu$, $e\tau$ or $\mu \tau$), using 3.2 fb$^{-1}$ of proton–proton collision data at $\sqrt{s}=13$ TeV collected in 2015 by the ATLAS detector at the Large Hadron Collider. No excess over the Standard Model prediction is observed. Limits at the 95 % credibility level are set on the mass of a $Z^\prime$ boson with lepton-flavour-violating couplings at 3.0, 2.7 and 2.6 TeV, and on the mass of a supersymmetric $\tau$ sneutrino with *R*-parity-violating couplings at 2.3, 2.2 and 1.9 TeV, for $e\mu$, $e\tau$ and $\mu \tau$ final states, respectively. The results are also interpreted as limits on the threshold mass for quantum black hole production.

## Introduction

Within the Standard Model (SM) of particle physics, direct production of lepton pairs with different flavours ($\ell \ell ^{\prime }$) is forbidden. However, lepton flavour violation (LFV) is allowed in many extensions of the SM. Models with additional gauge symmetries, e.g. production of a new heavy neutral gauge boson, similar to a $Z^\prime$ boson [1], scalar neutrinos in *R*-parity-violating (RPV) [2, 3] supersymmetry (SUSY) [4, 6–10], or low-scale gravity models predicting quantum black hole (QBH) production [11] can produce decays to lepton-flavour-violating final states. Processes leading to flavour-violating dilepton final states have a clear detector signature and a low background from SM processes. The Drell–Yan (DY) process (dilepton production in hadron–hadron collisions), an irreducible background for same-flavour dilepton searches, is limited to the production and decay of a ditau system, enhancing the sensitivity to a possible signal. This paper looks for final states with two leptons of different flavour in proton–proton (*pp*) collisions at $\sqrt{s}=13$ TeV. The invariant mass of the two leptons ($m_{\ell \ell ^{\prime }}$) is used as the search variable.

A common extension of the SM is the addition of an extra *U*(1) gauge symmetry resulting in a massive vector boson known as a $Z^\prime$ boson [1]. The search presented in this paper assumes a $Z^\prime$ boson that has the same fermion couplings as the SM $Z$ boson in the quark sector, but only leptonic decays that violate LFC are allowed. The addition of lepton-flavour-violating processes, $Z^\prime$ $\rightarrow e\mu$, $e\tau$, $\mu \tau$, requires new couplings between leptons of different generations: $Q^{\ell }_{\mathrm {12}}$, $Q^{\ell }_{\mathrm {13}}$ and $Q^{\ell }_{\mathrm {23}}$, where the subscripts denote lepton generations. For the model considered, this paper assumes $Q_{ij}^{\ell }$ equal to the SM $Z$ boson coupling to one lepton and only one LFV coupling different from zero at the same time. The ATLAS and CMS Collaborations have placed limits on the $e\mu$, $e\tau$ and $\mu \tau$ couplings as a function of the $Z^\prime$ boson mass up to 2.5 TeV, using the full $\sqrt{s}=8$ TeV [12, 13].

In RPV SUSY, the Lagrangian terms allowing LFV can be expressed as $\frac{1}{2}\lambda _{ijk}L_i L_j\bar{e_k} + \lambda ^{\prime }_{ijk}L_i Q_j\bar{d_k}$, where *L* and *Q* are the *SU*(2) doublet superfields of leptons and quarks, *e* and *d* are the *SU*(2) singlet superfields of leptons and down-like quarks, $\lambda$ and $\lambda ^\prime$ are Yukawa couplings, and the indices *i*, *j* and *k* denote fermion generations. A $\tau$ sneutrino ($\tilde{\nu }_{\tau }$) may be produced in *pp* collisions by $d\bar{d}$ annihilation and subsequently decay to $e\mu$, $e\tau$, or $\mu \tau$. Although only $\tilde{\nu }_{\tau }$ is considered in this paper, results apply to any sneutrino flavour. For the theoretical prediction of the cross-section times branching ratio, the $\tilde{\nu }_{\tau }$ coupling to first-generation quarks ($\lambda ^{\prime }_{311}$) is assumed to be 0.11 for all channels. As for the $Z^\prime$ model, only one decay to a lepton-flavour-violating final state is allowed at the same time. As such, for an $e\mu$ final state, it is assumed that $\lambda _{312}=\lambda _{321}=0.07$, for $e\tau \lambda _{313}=\lambda _{331}=0.07$ and $\mu \tau \, \lambda _{323}=\lambda _{332}=0.07$. These values are consistent with benchmark couplings used in previous ATLAS and CMS searches [12, 13]. The ATLAS Collaboration has placed limits up to 2.0 TeV on the mass of an RPV SUSY $\tilde{\nu }_{\tau }$ [12].

Various models introduce extra dimensions in order to lower the value of the Planck mass ($M_{\mathrm {P}}$) and solve the hierarchy problem. The search presented in this paper focuses on the ADD model [14], assuming $n=6$, where *n* is the number of extra dimensions, and the RS model [15], with one extra dimension. Due to the increased strength of gravity at short distances, *pp* collisions at the Large Hadron Collider (LHC) could produce states with masses beyond the threshold mass ($M_{\mathrm {th}}$), satisfying the Hoop conjecture [16] and form black holes. For the model considered, $M_{\mathrm {th}}$ is assumed to be equivalent to the extra-dimensional Planck scale. It is expected that, for masses beyond 3–5$M_{\mathrm {th}}$, thermal black holes would be produced [17, 18], characterised by high-multiplicity final states. As such, for the search presented in this paper, it is more interesting to focus on the mass region below 3–5$M_{\mathrm {th}}$, known as the quantum gravity regime, investigated in Refs. [19–21]. Non-thermal (or quantum) black holes would be formed in this region, and could decay to two-particle final states, producing the topology this analysis is focused on. Such quantum black holes would form a continuum in mass from $M_{\mathrm {th}}$ up to the beginning of the thermal regime. For the model considered in this paper, the thermal regime is assumed to start at 3$M_{\mathrm {th}}$. The decay of quantum black holes would be governed by a yet unknown theory of quantum gravity. The two main assumptions of the extra-dimensions models considered [11] in this paper are:

- gravity couples with equal strength to all SM particle degrees of freedom;
- gravity conserves local symmetries (colour, electric charge) but can violate global symmetries such as LFC and baryon number conservation.

Following these assumptions, the branching ratio (BR) to each final state can be calculated. Two initial states could give rise to a quantum black hole decaying into a lepton-flavour-violating final state: $q\bar{q}$ and *gg*. The branching ratio to $\ell \ell ^{^{\prime }}$ is 0.87 % (0.34 %) for a $q\bar{q}$ (*gg*) initial state [11]. This model was used in previous ATLAS and CMS searches in dijet [22–24], lepton+jet [25], photon+jet [26], $e\mu$ [13] and same-flavour dilepton [27] final states.

## The ATLAS detector

The ATLAS detector [28] is a general-purpose particle detector with approximately forward-backward symmetric cylindrical geometry.^1^ It is composed of four main components, each responsible for identifying and reconstructing different types of particles: the inner detector (ID), the electromagnetic and hadronic calorimeters, and the muon spectrometer (MS). Each of the sub-detectors is divided into two components, barrel and endcap, to provide coverage close to $4\pi$ in solid angle. In addition, two magnet systems are in place to allow charge and momentum measurements: an axial magnetic field of 2.0T provided by a solenoid surrounding the ID, and a toroidal magnetic field for the MS. The ID, the component of the ATLAS detector closest to the interaction point, reconstructs the trajectories of charged particles in the region $|\eta |<2.5$ and measures their momenta. It is composed of three sub-systems:

i) a silicon pixel detector, including the newly installed insertable B-layer [29, 30];
ii) the semi-conductor tracker, used in conjunction with the silicon pixel detector to determine primary and secondary vertices with high precision thanks to their high granularity;
iii) the transition radiation tracker, providing additional tracking in the region $|\eta |<2.0$ and electron identification.

Surrounding the ID, lead/liquid-argon (LAr) sampling calorimeters provide electromagnetic (EM) energy measurements with high granularity. A steel/scintillator-tile hadronic calorimeter covers the central pseudorapidity range ($|\eta | <$ 1.7). The endcap and forward regions are LAr calorimeters with copper or tungsten absorbers for both the EM and hadronic energy measurements up to $|\eta | <$ 4.9. Built around the calorimeter system, the MS is the sub-detector furthest from the interaction point. It consists of three layers of precision tracking chambers and fast detectors for triggering on muons. Tracking coverage is provided up to $|\eta | <$ 2.7 through the use of monitored drift tubes and, in the innermost layer, cathode strip chambers for $|\eta | >$ 2.0, while trigger coverage is provided by resistive plate and thin gap chambers up to $|\eta | <$ 2.4.

The trigger and data-acquisition system is based on two levels of online event selection [31]: the level-1 trigger and the high-level trigger. The level-1 trigger is hardware-based and uses a subset of detector information to provide quick trigger decisions and reduce the accepted rate to 100 kHz. The high-level trigger is software-based and exploits the full detector information to further reduce the accepted rate to about 1 kHz.

## Data and Monte Carlo simulated samples

The data sample used for this analysis was collected with the ATLAS detector during the 2015 LHC run with *pp* collisions at a centre-of-mass energy of 13 TeV with a 25 ns minimum proton bunch spacing. After selecting periods with stable beams and requiring all detector systems to be fully functional, the total integrated luminosity for the analysis is 3.2 fb$^{-1}$. The uncertainty in the integrated luminosity is 5.0 %. It is derived following a methodology similar to that detailed in Ref. [32], from a calibration of the luminosity scale using *x*–*y* beam-separation scans performed in August 2015.

The $pp\rightarrow Z^\prime \rightarrow \ell \ell ^{\prime }$ signal samples are generated at leading order (LO) using the Monte Carlo (MC) generator Pythia 8.186 [33] with the NNPDF23LO [34] parton distribution function (PDF) set and the A14 [35] set of tuned parameters (tune). Signal samples with 25 mass points ranging from 0.5 TeV up to 5 TeV are generated in 0.1 TeV steps from 0.5 to 2.0 TeV, 0.2 TeV steps from 2.0 to 3.0 and 0.5 TeV steps from 3.0 to 5.0 TeV. The production cross-section is calculated with the same MC generator used for simulation. No mixing with the SM $Z$ boson is included.

The $d\bar{d}\rightarrow \tilde{\nu }_{\tau }\rightarrow \ell \ell ^{\prime }$ signal samples are generated at LO using the MC generator MG5_aMC@NLO v2.3.3 [36] interfaced to the Pythia 8.186 parton shower model with the NNPDF23LO PDF set and the A14 tune. The signal samples are generated at the same pole-masses as for the $Z^\prime$ described above. The cross-section is calculated at LO with the same MC generator used for simulation. A next-to-leading order (NLO) correction factor (*K*-factor) is calculated for the cross-section based on Ref. [37] using LoopTools v2.2 [38].

The $pp\rightarrow$QBH$\rightarrow \ell \ell ^{\prime }$ samples are generated with QBH 3.00 [39] using the CTEQ6L1 [40] PDF set and the A14 tune, for which Pythia 8.183 provides showering and hadronisation. For each extra-dimensional model, eleven $M_{\mathrm {th}}$ points in 0.5 TeV steps were produced: from 3.0 to 8.0 TeV for the ADD $n=6$ model, and from 1.0 to 6.0 TeV for the RS $n=1$ model. The production cross-section is calculated with the same MC generator used for simulation. These two models have differences in the number and nature of the additional extra dimensions (large extra dimensions for ADD, one highly warped extra dimension for RS). In particular, the ADD model allows production of black holes with a larger gravitational radius and hence the parton–parton cross-section for this model is larger than for the RS model. Therefore, the $M_{\mathrm {th}}$ range of the generated samples is different for the two models.

The SM background to the LFV dilepton search is composed of several processes which can produce a final state with two different-flavour leptons. The dominant background contributions originate from $t\bar{t}$ and single-top production, with the subsequent decays of the top quark producing leptonically decaying $W$ bosons. Other backgrounds originate from diboson ($WW$, $WZ$ and $ZZ$) production and the DY process, $q\bar{q}\rightarrow Z/\gamma ^{*}\rightarrow \tau \tau$, which can produce different-flavour final states through the leptonic decay of the $W$ and $Z$ bosons and the $\tau$ lepton. Multi-jet and $W$+jets processes contribute due to the misidentification of jets as leptons.

Backgrounds from top quark production include $t\bar{t}$ and single-top with an associated $W$ boson ($tW$). Both the $t\bar{t}$ and single-top-quark backgrounds are generated at NLO using the Powheg-Box v2 [41] generator with the CT10 [42] PDF set in the matrix element (ME) calculations. Pythia 6.4.28 [43] and the corresponding Perugia 2012 tune [44] are used to simulate the parton shower, hadronisation, and the underlying event. Top quarks are decayed using MadSpin [45], preserving all spin correlations. The parameter which controls the $p_{\mathrm {T}}$ of the first emission beyond the Born configuration in Powheg, called *hdamp*, is set to the mass of the top quark. The main effect of this is to regulate the high-$p_{\mathrm {T}}$ emission against which the $t\bar{t}$ system recoils. The mass of the top quark is set to 172.5GeV. A value of 831$^{+20}_{-29}$ (scale) $^{+35}_{-35}$ (PDF+$\alpha _{\mathrm {S}}$) $^{+23}_{-22}$ (mass uncertainty) pb is used for the $t\bar{t}$ production cross-section, computed with Top++ 2.0 [46], incorporating next-to-next-to-leading order (NNLO) corrections in QCD, including resummation of next-to-next-to-leading logarithmic (NNLL) soft gluon terms. A $tW$ production cross-section of $71.7\pm 3.8$ pb is used, as computed in Ref. [47] to approximately NNLO (NNLL+NLO) accuracy.

Diboson processes with four charged leptons, three charged leptons and one neutrino, two charged leptons and two neutrinos, or one boson decaying to leptons and the other hadronically, are simulated using the Sherpa 2.1.1 generator [48]. The matrix elements contain all diagrams with four electroweak vertices. Fully-leptonic decays are calculated for up to one (four leptons, two leptons and two neutrinos) or zero partons (three leptons and one neutrino) at NLO and up to three partons at LO using the Comix [49] and OpenLoops [50] ME generators and merged with the Sherpa parton-shower [51] using the ME+PS@NLO prescription [52]. Semileptonic decays are calculated for up to one ($ZZ$) or zero ($WW$, $WZ$) additional partons at NLO and up to three additional partons at LO using Comix and OpenLoops. The CT10 PDF set is used in conjunction with the default parton-shower tuning provided by the Sherpa authors in the release.

The Drell–Yan process is generated at LO using the Pythia8 MC generator with the NNPDF23LO PDF set. The same generator is used for showering and hadronisation. Dilepton mass-dependent *K*-factors are applied to account for higher-order QCD and electroweak corrections and to normalise the cross-section to NNLO, computed using FEWZ 3.1 [53] and the CT14NNLO PDF set [54].

SM processes such as $W$+jets and multi-jet production involving jets that fake leptons are evaluated through the use of data-driven methods detailed in Sect. 5. The $W$+jets contribution is estimated with the aid of Sherpa MC simulated samples. Matrix elements are calculated for up to two partons at NLO and four partons at LO using the same procedures, prescriptions and PDF set adopted for the diboson samples. The $W$+jets events are normalised to the NNLO cross-section [55].

For all samples used in this analysis, the effects of multiple interactions per bunch crossing (pile-up) are accounted for by overlaying minimum-bias events simulated with Pythia8 and re-weighting the MC events to reproduce the distribution of the average number of interactions per bunch crossing observed in the data. The MC generated events were processed with the ATLAS simulation infrastructure [56], based on Geant4 [57], and passed through the trigger simulation and the same reconstruction software used for the data.

## Object and event selection

Candidate muon tracks are initially reconstructed independently in the ID and the MS. The two tracks are then used as input to a combined fit which takes into account the energy loss in the calorimeter and multiple scattering. Muon identification is based on information from both the ID and MS to ensure that muons are reconstructed with the optimal momentum resolution up to very high $p_{\mathrm {T}}$ using the *High*-$p_{\mathrm {T}}$ operating point [58]. Muon candidates with hits in regions of the MS with residual misalignments, such as the barrel–endcap overlap region ($1.01<|\eta |<1.1$), are vetoed. Muon tracks are required to be within the ID acceptance region^2^ of $|\eta |<2.5$ and have at least three hits in each of the three traversed precision chambers in the MS. An exception is made in the region $|\eta |<0.1$ due to the MS gap in that region, where tracks with at least three hits in a single precision chamber are allowed. In order to suppress hadrons misidentified as muons, the momentum measurements of the ID and the MS must agree within seven standard deviations. As well as the quality cuts, muon candidates must fulfil $p_{\mathrm {T}}>65$ GeV and transverse impact parameter ($d_{\mathrm {0}}$) significance $|d_{\mathrm {0}}/\sigma _{d_{\mathrm {0}}}|<3$ with respect to the beam line, where $\sigma _{d_{\mathrm {0}}}$ is the uncertainty in the value of the transverse impact parameter. The distance between the *z*-position of the point of closest approach of the muon track in the ID to the beamline and the *z*-coordinate of the primary vertex^3^ ($\Delta z_{\mathrm {0}}$) is required to satisfy $|\Delta z_{\mathrm {0}}\sin \theta |<0.5$ mm. This requirement aims to reduce the background from cosmic rays and from muons originating from heavy-flavour decays. Moreover, candidates are required to fulfil track-based isolation criteria with a fixed efficiency of 99 % over the full range of muon momentum to further reduce contamination from non-prompt muons. The sum of the transverse momentum of tracks in an isolation cone of size $\Delta R=0.2$ (excluding the muon itself) divided by the muon $p_{\mathrm {T}}$ is used as a discrimination criterion for the track-based isolation.

Electron candidates are formed from the energy in clusters of cells in the electromagnetic calorimeter associated with a track in the ID [59]. A multivariate analysis approach, employing a likelihood (LH) discriminant, is built to suppress contributions from hadronic jets, photon conversions, Dalitz decays and semileptonic heavy-flavour hadron or kaon decays. The LH discriminant utilises lateral and longitudinal calorimeter shower shape, tracking and cluster–track matching quantities. The discriminant criterion is a function of the tranverse momentum and $|\eta |$ of the candidate electron. Two operating points are used in this analysis, as defined in Ref. [60]: *Medium* and *Tight*. The *Tight* working point (90 % efficient at $p_{\mathrm {T}}=65$ GeV) is required for electron candidates, while the *Medium* working point (95 % efficient at $p_{\mathrm {T}}=65$ GeV) is used to estimate the background contribution from jets misidentified as electrons (as discussed in Sect. 5). Electron candidates must fulfil $p_{\mathrm {T}}>65$ GeV and $|\eta |<2.47$, excluding the region $1.37<|\eta |<1.52$, where the energy reconstruction performance is degraded due to the presence of extra inactive material. Further requirements are made on the impact parameter: $|d_{\mathrm {0}}/\sigma _{d_{\mathrm {0}}}|<5$ and $|\Delta z_{\mathrm {0}}\sin \theta |<0.5$ mm. To reject electrons faked by muons, electron candidates within a $\Delta R=0.2$ cone around a muon candidate are removed. Moreover, candidates are required to fulfil relative track- (as defined above for muon candidates) and calorimeter-based isolation requirements with a fixed efficiency of 99 %, to suppress background from non-prompt leptons originating from heavy-flavour or kaon decays, charged hadrons and photon conversions from $\pi ^{\mathrm {0}}$ decays. The sum of the calorimeter transverse energy deposits in an isolation cone of size $\Delta R=0.2$ (excluding the electron itself) divided by the electron $p_{\mathrm {T}}$ is used as a discrimination criterion for the calorimeter-based isolation.

Jets, used in the reconstruction of hadronically-decaying $\tau$ leptons, are reconstructed using the anti-$k_{t}$ algorithm [61] with a radius parameter (*R*) of 0.4, using as input topological clusters [62] of calorimeter cells [63]. The three-dimensional topological clusters are built from topologically connected calorimeter cells that contain a significant signal above noise. The cluster energies are corrected for inactive material and out-of-cluster energy losses. Jet calibrations derived from $\sqrt{s}=13$ TeV simulation, and collision data taken at $\sqrt{s}=8$ and $\sqrt{s}=13$ TeV, are used to correct the jet energies and directions to those of the particles from the hard-scatter interaction. This calibration procedure, described in Refs. [63–65], is improved by a data-derived correction to the relative calibration of jets in the central and the forward regions.

The reconstruction of $\tau$ leptons and their visible hadronic decay products, referred to as $\tau _{\mathrm {had}}^{\mathrm {vis}}$, starts with jets reconstructed from topological clusters as described above. Hadronic decays of $\tau$ leptons ($\tau _{\mathrm {had}}$) are mainly characterised by the presence of one or three charged particles, accompanied by a neutrino and possibly other neutral particles [66]. The $\tau _{\mathrm {had}}^{\mathrm {vis}}$ candidates must have energy deposits in the calorimeters in the range $|\eta |<2.5$, with the transition region between the barrel and endcap calorimeters ($1.37<|\eta |<1.52$) excluded, a transverse momentum greater than 40GeV, one or three associated tracks and an electric charge of ±1. Their identification is performed using a multivariate algorithm that employs boosted decision trees (BDTs) to discriminate against quark- and gluon-initiated jets using shower shape and tracking information. An additional dedicated likelihood-based veto is used to reduce the number of electrons misidentified as $\tau _{\mathrm {had}}$. The $\tau$ lepton candidates which overlap with electron or muon candidates within a cone of $\Delta R=0.2$ are rejected.

The event selection requires a single-muon or single-electron trigger with a $p_{\mathrm {T}}$ threshold of 50 GeV for muons, and 60 or 120 GeV for electrons. The single-electron trigger with higher $p_{\mathrm {T}}$ threshold has a looser LH identification requirement, resulting in an increased trigger efficiency at high $p_{\mathrm {T}}$. Selected events must have a reconstructed primary vertex and exactly two different-flavour lepton candidates meeting the above-mentioned criteria. Events with an additional lepton or extra “loose” lepton^4^ are vetoed. Moreover, the lepton candidates have to be back-to-back in the $\phi$ direction with $\Delta \phi (\ell ,\ell ^{\prime })>2.7$. No requirement is made on the respective charges of the leptons as it is found to reduce the signal efficiency by as much as 6 % for the highest-mass signals considered due to charge mis-assignment, without a significant effect on the background rejection. For a $Z^\prime$ boson with a mass of 1.5 TeV, the acceptance times efficiency^5^ ($A\epsilon$) of the selection requirements is approximately 50, 25 and 20 % for the $e\mu$, $e\tau$ and $\mu \tau$ final states, respectively. To account for differences between data and simulation, corrections are applied to the lepton trigger, reconstruction, identification, and isolation efficiencies as well as the lepton energy/momentum resolution and scale [58, 59, 66].

The missing transverse momentum ($E_{\mathrm {T}}^{\mathrm {miss}}$) is defined as the negative vector sum of the transverse momenta of all identified physics objects (electrons, photons [67], muons, taus, jets) and an additional soft term. The soft term is constructed from all tracks that are associated with the primary vertex but not with any physics object. In this way, the missing transverse momentum is adjusted for the best calibration of the jets and the other identified physics objects above, while maintaining pile-up independence in the soft term [68].

An additional variable to estimate the contribution from reducible backgrounds is used: the transverse mass ($m_{\mathrm {T}}$) of a lepton and the $E_{\mathrm {T}}^{\mathrm {miss}}$, defined as:

1 $$\begin{aligned} m_{\mathrm {T}} = \sqrt{2 p_{\mathrm {T}}E_{\mathrm {T}}^{\mathrm {miss}} (1-\cos (\Delta \phi (\ell ,E_{\mathrm {T}}^{\mathrm {miss}}))}\;, \end{aligned}$$

where $\Delta \phi (\ell ,E_{\mathrm {T}}^{\mathrm {miss}})$ is the azimuthal angle between the lepton $p_{\mathrm {T}}$ and $E_{\mathrm {T}}^{\mathrm {miss}}$ direction.

For events in the $e\tau$ and $\mu \tau$ channels, in order to reconstruct the dilepton invariant mass more accurately, the neutrino four-momentum is taken into account. The hadronic decay of a $\tau$ lepton from a heavy resonance leads to the neutrino and the resultant jet being nearly collinear. The neutrino four-momentum is reconstructed from the magnitude of the missing transverse momentum, and is assumed to be collinear with the $\tau _{\mathrm {had}}$ candidate. For the mentioned channels, the above technique significantly improves the mass resolution and search sensitivity.

## Background estimation

The background processes for this search can be divided into two categories: irreducible and reducible backgrounds. The former is composed of processes which can produce two different flavour prompt leptons in the final state, including the DY$\rightarrow \tau \tau$ process, $t\bar{t}$, single top, and diboson production. These processes are modelled using MC simulated samples. Reducible backgrounds occur when jets are mis-reconstructed as leptons, and require the use of data-driven techniques.

The MC samples used to estimate single-top and $t\bar{t}$ production are statistically limited for dilepton invariant masses above 1 TeV. Therefore, fits to the $m_{\ell \ell ^{\prime }}$ distribution using monotonically decreasing functions are used to extrapolate those backgrounds to the region $m_{\ell \ell ^{\prime }}>1$ TeV. Two functional forms are investigated, chosen for their stability when varying the fit range and for the quality of the fit:

2 $$\begin{aligned} \mathrm {e}^{-a} \cdot m_{\ell \ell ^{\prime }}^b \cdot m_{\ell \ell ^{\prime }}^{c\cdot \mathrm {ln}(m_{\ell \ell ^{\prime }})} \quad \mathrm {and} \quad \frac{a}{(m_{\ell \ell ^{\prime }}+b)^c}\;, \end{aligned}$$

where *a*, *b* and *c* are free parameters in the fit. A study of the stability of the fit was performed by varying the lower and upper limits of the fit range between 200–300 GeV and 1000–1200 GeV in 25 GeV steps, respectively. The stitching point between the MC estimation and the fit is chosen to be at 900 GeV for the top quark background. The nominal extrapolation is then taken to be the median of all the tested fit ranges using both functional forms. Good agreement is found between the fit prediction and the available MC events. The addition in quadrature of the fit parameter uncertainties and the RMS of all fit variations is assigned as a systematic uncertainty.

The contribution from reducible backgrounds originate mainly from $W$+jets and multi-jet processes. The background of muons originating from hadronic decays is found to be negligible compared to the contribution from fake electrons and taus. Therefore, in the $e\mu$ channel, where the contribution of the reducible background is expected to be small, these non-prompt muons are neglected. The reducible background in that channel is then reduced to events with one prompt muon and a jet faking an electron. This background contribution is usually not well modelled by MC simulation.

For the $e\mu$ channel, a technique known as the matrix method, described in Ref. [27], is employed. Exclusive samples are defined by loosening the selection criteria for electron candidates. Here the matrix method involves two parameters that need to be determined as a function of electron $p_{\mathrm {T}}$: the probability of a loose electron to pass the full object selection, the so-called *real electron efficiency* ($\epsilon _{\mathrm {R}}$), and the probability of a jet fulfilling the loose electron selection criteria to pass the full selection, known as the *electron fake rate* ($\epsilon _{\mathrm {F}}$). The former is evaluated from MC simulation, while the latter is evaluated in a data sample dominated by multi-jet events. To construct this multi-jet control sample, it is required that $E_{\mathrm {T}}^{\mathrm {miss}}<25$ GeV and $m_{\mathrm {T}}<50$ GeV in order to suppress the $W$+jets contribution. Contamination from $W$+jets and other SM background processes (top, diboson, and $Z\rightarrow \ell \ell$) is subtracted using MC predictions.

For the $e\tau$ and $\mu \tau$ channels, the $\tau$ fake rate is measured in data in a $W$ $\rightarrow e$/$\mu$+jets control region as a function of the $\tau _{\mathrm {had}}^{\mathrm {vis}}$ $p_{\mathrm {T}}$. The region is defined to be orthogonal to the signal selection by reversing the $\Delta \phi (\ell ,\ell ^{\prime })$ requirement. Only events with exactly one electron or muon fulfilling all selection criteria (as defined in Sect. 4), as well as $m_{\mathrm {T}}>60$ GeV, are used. The $\tau _{\mathrm {had}}$ candidates present in those events are dominated by jets. The $\tau$ fake rate is defined as the fraction of jets fulfilling all $\tau$ object selection criteria, including the multivariate BDT-based identification. The derived fake rate is used to weight simulated $W$+jets events. After obtaining the fake-rate-weighted $m_{\ell \ell ^{\prime }}$ distribution, a normalisation factor for the $W$+jets background is obtained in a $W$+jets enriched region to scale the overall normalisation of the MC simulation to that of the data. The $W$+jets enriched region is defined as a sub-set of the signal selection by further requiring $E_{\mathrm {T}}^{\mathrm {miss}}>30$ GeV and lepton $p_{\mathrm {T}}<150$ GeV to avoid possible signal contamination. The contribution from events with an electron/muon and a fake $\tau _{\mathrm {had}}$ is found to make up around 55 % of the overall background in the $e\tau$ and $\mu \tau$ channels.

To evaluate the fake background from events with a real $\tau _{\mathrm {had}}$ and a fake electron/muon in the $e\tau$ and $\mu \tau$ channels, a fake-electron/muon enriched sample is defined by requiring a non-isolated electron/muon and a $\tau _{\mathrm {had}}$ candidate. Three regions are defined:

- **Region 1:** pairs of a non-isolated electron/muon and a $\tau _{\mathrm {had}}$ with the same electric charge;
- **Region 2:** pairs of an isolated electron/muon and a $\tau _{\mathrm {had}}$ with the same electric charge;
- **Region 3:** all pairs of a non-isolated electron/muon and a $\tau _{\mathrm {had}}$.

The $m_{\ell \ell ^{\prime }}$ shape of the contribution is obtained from region 3 by subtracting the contribution from other background sources to the data, while the ratio of isolated to non-isolated leptons in regions 1 and 2 is used to normalise this background contribution appropriately. The contribution from events with a fake electron/muon and a real $\tau$ lepton is found to be below 1 % in the $\mu \tau$ channel, while in the $e\tau$ channel its contribution to the overall SM background is close to 5 %.

A summary of the contribution from each SM background in each of the final states can be found in Sect. 8.

## Systematic uncertainties

Sources of systematic uncertainty are divided in two categories: theoretical and experimental. Uncertainties in the predicted cross-section times branching ratio and the modelling of the $m_{\ell \ell ^{\prime }}$ shape of the background processes considered are regarded as theoretical uncertainties, while uncertainties relating to the simulation of the detector response are regarded as experimental uncertainties. Theoretical uncertainties (such as PDF-related uncertainties) in the signal cross-section are not considered in this paper.

The PDF uncertainties are the dominant theoretical systematic uncertainties, together with the uncertainty of the extrapolation to estimate the background contribution at high-mass (as described in Sect. 5). The contribution from PDF uncertainties is estimated using different PDF sets and eigenvector uncertainty sets within a particular PDF. The CT10 PDF uncertainty due to eigenvector variations is evaluated through the use of LHAPDF [69] following the prescriptions outlined in Ref. [70]. The uncertainty related to the choice of PDF is evaluated by comparing the results with those from the central value of other PDF sets such as MMHT2014 [71], NNPDF3.0 [72] and CT14 [54]. PDF-related uncertainties in the signal shape are not considered. The uncertainties in the $m_{\ell \ell ^{\prime }}$ modelling in $t\bar{t}$ events is obtained using separate MC samples generated with variations in the renormalisation and factorisation scales and the *hdamp* parameter (as defined in Sect. 3).

The effect of experimental systematic uncertainties is assessed through the uncertainties associated to the corrections applied to simulated processes, including lepton momentum resolution and scale, and trigger, identification, reconstruction and isolation efficiencies [58, 59, 66]. The efficiencies are evaluated using events from the $Z\rightarrow \ell \ell$ peak and then extrapolated to high energies.

Mismodelling of the muon momentum resolution at the TeV scale, such as due to residual misalignment of the muon precision chambers, can alter the signal and background shapes. An uncertainty related to this is obtained from studies performed in dedicated data-taking periods with no magnetic field in the MS. The muon reconstruction efficiency is affected at high-$p_{\mathrm {T}}$ by possible large energy losses in the calorimeter. The associated uncertainty is estimated by comparing studies with $Z\rightarrow \mu \mu$ events in data extrapolated at high-$p_{\mathrm {T}}$ to the results predicted by MC simulation [73]. The effect on the muon reconstruction efficiency was found to be approximately 3 % per TeV as a function of muon $p_{\mathrm {T}}$.

The uncertainty in the electron identification efficiency extrapolation is based on the differences in the electron shower shapes in the EM calorimeters between data and MC simulation in the $Z\rightarrow ee$ peak, which are propagated to the high-$p_{\mathrm {T}}$ electron sample. The effect on the electron identification efficiency was found to be 2 % and is independent of $p_{\mathrm {T}}$ for electrons with transverse momentum above 150GeV [73].

The treatment of systematic uncertainties for $\tau$ leptons with $p_{\mathrm {T}}$ up to 100 GeV is detailed in Ref. [66]. An additional uncertainty of 20 % per TeV is assigned to the reconstruction efficiency of $\tau$ leptons with pT > 100 GeV to account for the the degradation of the modelling and reconstruction efficiency due to track merging, derived through studies in simulation and in dijet data events at 8 TeV [74].

The uncertainties associated to the matrix method used for the $e\mu$ channel are evaluated by considering effects on the $\epsilon _{\mathrm {F}}$ measurement, including the multi-jet control sample definition and the uncertainties in the overall normalisation. The former effect is evaluated by shifting the $E_{\mathrm {T}}^{\mathrm {miss}}$ and $m_{\mathrm {T}}$ requirements by ±10 GeV, while the latter is taken into account by varying the MC subtraction of other SM processes by the luminosity and experimental systematic uncertainties. For the $e\tau$ and $\mu \tau$ channels, the uncertainty in the $\tau$ fake rate and $W$+jets normalisation in the MC subtraction is considered. The $\tau$ fake rate is re-evaluated when removing the $m_\mathrm {T}$ requirement, requiring $m_{\tau \ell }>110$ GeV to reduce the Drell–Yan background and vetoing events with a jet identified as originating from a *b*-quark [75] to reduce top-quark background contamination. The variations obtained for the $\tau$ fake rates are assigned as systematic uncertainties. Given the limited data available for $\tau$ lepton $p_{\mathrm {T}}>500$ GeV, the statistical uncertainty from the last data bin is used together with an uncertainty of 20 % per TeV in $\tau$ lepton $p_{\mathrm {T}}$. The uncertainty on the $W$+jets normalisation is obtained by recalculating the normalisation factor after a variation for each of the experimental systematic uncertainties outlined in Table 1.

The uncertainty in the reducible background estimate is found to be close to 50, 30 and 40 % for the $e\mu$, $e\tau$ and $\mu \tau$ channels, respectively, at $m_{\ell \ell ^{\prime }}=1.0$ TeV and it is of comparable size to the PDF uncertainty in the $e\tau$ and $\mu \tau$ channels. However, the contribution from reducible backgrounds in the $e\mu$ channel is below 10 %, while for $e\tau$ and $\mu \tau$ final states it is the leading background together with the contribution from top quark production.

Experimental systematic uncertainties common to signal and background processes are assumed to be correlated. The effect of systematic uncertainties on the estimated SM background yields is summarised in Table 1.

For signal processes, only experimental systematic uncertainties are considered. The statistical uncertainty of the signal MC samples is 3 %.

**Table 1:** Quantitative summary of the systematic uncertainties taken into account for background processes. Values are provided for $m_{\ell \ell ^{\prime }}$ values of 1, 2 and 3 TeV. The statistical error includes the extrapolation uncertainties of the top quark background in the high-$m_{\ell \ell ^{\prime }}$ region together with the uncertainty related to the number of MC events. Uncertainties are quoted with respect to the total background. N/A means the systematic uncertainty is not applicable. The expected SM background in a mass window within $\pm 0.1\cdot m_{\ell \ell ^{\prime }}$ is also reported

Source	$m_{\ell \ell ^{\prime }}=1$ TeV			$m_{\ell \ell ^{\prime }}=2$ TeV			$m_{\ell \ell ^{\prime }}=3$ TeV		
	$e\mu$	$e\tau$	$\mu \tau$	$e\mu$	$e\tau$	$\mu \tau$	$e\mu$	$e\tau$	$\mu \tau$
PDF uncertainty	17 %	15 %	15 %	35 %	38 %	35 %	70 %	75 %	70 %
Luminosity	5 %	5 %	5 %	5 %	5 %	5 %	5 %	5 %	5 %
Statistical	18 %	11 %	15 %	80 %	27 %	27 %	120 %	28 %	30 %
Reducible background	5 %	29 %	40 %	5 %	35 %	75 %	5 %	45 %	85 %
Top quark production modelling	5 %	3 %	4 %	12 %	4 %	5 %	15 %	10 %	8 %
Electron trigger efficiency	1 %	1 %	N/A	1 %	1 %	N/A	1 %	1 %	N/A
Electron identification	2 %	2 %	N/A	2 %	2 %	N/A	2 %	2 %	N/A
Electron energy scale and resolution	3 %	3 %	N/A	3 %	3 %	N/A	3 %	3 %	N/A
Muon reconstruction efficiency	2 %	N/A	2 %	4 %	N/A	4 %	6 %	N/A	6 %
Muon scale and resolution	4 %	N/A	4 %	12 %	N/A	12 %	20 %	N/A	20 %
Muon trigger efficiency	2 %	N/A	2 %	2 %	N/A	2 %	2 %	N/A	2 %
Tau identification	N/A	4 %	4 %	N/A	5 %	5 %	N/A	6 %	6 %
Tau reconstruction	N/A	3 %	3 %	N/A	4 %	4 %	N/A	4 %	4 %
Tau energy calibrations	N/A	2 %	2 %	N/A	3 %	3 %	N/A	4 %	4 %
Total	27 %	35 %	44 %	90 %	59 %	90 %	140 %	90 %	120 %
SM background in $m_{\ell \ell ^{\prime }}\pm 0.1\cdot m_{\ell \ell ^{\prime }}$	3.9	11.9	11.4	0.09	0.55	0.49	0.002	0.014	0.017

## Statistical analysis

If no deviations from the SM prediction are observed, model-dependent exclusion limits are extracted using a Bayesian method and implemented with the software package Bayesian Analysis Toolkit (BAT) [76] using a template shape method. A binned likelihood function ($\mathcal {L}$) is built as the product of the Poisson probability of observing $n_{\mathrm {obs}_k}$ when expecting $\mu _{k}$ in each of the mass bins used for the search:

3 $$\mathcal {L}(n_{\mathrm {obs}}| \theta ,\hat{\Omega }) = \prod ^{N_{\mathrm {bins}}}_{k=1} \dfrac{\mu ^{n_{\mathrm {obs}_k}}_{k}\mathrm {e}^{\mu _{k}}}{n_{\mathrm {obs}_{k}}!} \prod _{i=1}^{N_{\mathrm {Sys}}} G(\Omega _{i},0,1)\;,$$

where $\mu _{k}$ is the expected number of background and signal events ($\mu _{k}=N_{\mathrm {bkg}_k} + N_{\mathrm {sig}_k}(\theta )$) as a function of the parameter of interest $\theta$, $\hat{\Omega }$ is the vector of nuisance parameters introduced to account for the effect of systematic uncertainties in the expected yields, $N_{\mathrm {bins}}$ is the number of dilepton invariant mass bins, $N_{\mathrm {Sys}}$ is the total number of nuisance parameters and $G(\Omega _{i},0,1)$ is a Gaussian distribution with zero mean and unit standard deviation assumed to be the probability density function for the nuisance parameter $\Omega _{i}$. The dependence on the vector of nuisance parameters is removed through the use of a Markov Chain Monte Carlo integration technique. Bayes theorem is then applied to construct a posterior probability density function for the number of signal events assuming a uniform prior in the parameter of interest ($P(\theta )$). The number of signal events can be expressed in terms of the cross-section times branching ratio of the signal process ($\sigma \cdot \mathrm {BR}(X\rightarrow \ell \ell ^{\prime })$) as:

4 $$\begin{aligned} N_{\mathrm {sig}} = \sum ^{\mathrm {N_{\mathrm {bins}}}}_{k=1} N_{\mathrm {sig}_{k}} = \sigma \cdot \mathrm {BR}(X\rightarrow \ell \ell ^{\prime })\cdot L \cdot A\epsilon (X\rightarrow \ell \ell ^{\prime })\;, \end{aligned}$$

where *L* is the integrated luminosity of the dataset and $A\epsilon (X\rightarrow \ell \ell ^{\prime })$ is the acceptance times efficiency of the physics model tested. As such, a posterior probability density function is obtained for the signal $\sigma \cdot \mathrm {BR}$. A 95 % credibility level (CL) upper limit is obtained on the signal cross-section times branching ratio by finding the value of $\theta ^{\mathrm {95}}$ satisfying:

5 $$\begin{aligned} 0.95 = \dfrac{\int ^{\theta ^{\mathrm {95}}}_{\mathrm {0}} \mathcal {L^{\prime }}(n_{\mathrm {obs}}|\theta )P(\theta )\mathrm {d}\theta }{\int ^{\infty }_0\mathcal {L^{\prime }}(n_{\mathrm {obs}}|\theta )P(\theta ) \mathrm {d}\theta }\;, \end{aligned}$$

where $P(\theta )$ is the uniform prior probability mentioned above and $\mathcal {L^{\prime }}$ is the marginalised likelihood, obtained after performing the Markov Chain Monte Carlo integration over $\hat{\Omega }$. Expected exclusion limits are obtained by running 1000 pseudo-experiments (PE) for each of the signal mass points tested. The median value of the 95 % CL upper Bayesian limit PE distribution is taken as the expected limit. The one- and two-standard deviation intervals of the expected limit are obtained from the 1000 PE ensemble by finding the 68 and 95 % CL interval envelopes, respectively.

The predicted width of the $Z^\prime$ boson, 3 % for $m_{Z^\prime }=2$ TeV, is lower than the detector resolution for the $e\mu$ and the $\mu \tau$ channels, which are approximately 8 % and 12 %, respectively, at the same $Z^\prime$ boson mass. For the $e\tau$ final state the detector resolution is 4 % at $m_{Z^\prime }=2$ TeV, comparable to the $Z^\prime$ boson width. The width of the $\tilde{\nu }_{\tau }$ is below 1 % and hence the resolution of the detector is larger than the width for each of the final states investigated. For limit setting on the signal models investigated, a logarithmic $m_{\ell \ell ^{\prime }}$ binning is used with 40 mass bins between 120 and 10,000 GeV. The bin width is around 10 % in dilepton mass throughout the whole range.

## Results

Table 2 summarises the expected and observed yields in the validation and search regions for each of the channels considered in this search. The region $m_{\ell \ell ^{\prime }}<600$ GeV is defined as the validation region where the data is used to check the SM background prediction, while the region $m_{\ell \ell ^{\prime }}>600$ GeV is defined as the search region. Selected $e\mu$ events are dominated by $t\bar{t}$ events, while $W$+jets events are dominant for the $e\tau$ and $\mu \tau$ final states.

**Table 2:** Observed and expected numbers of (a) $e\mu$ , (b) $e\tau$, and (c) $\mu \tau$ events in the validation ($m_{\ell \ell ^{\prime }}<600$ GeV) and search regions ($m_{\ell \ell ^{\prime }}>600$ GeV) for the SM backgrounds and the signal models considered. The quoted errors include statistical and systematic uncertainties. The uncertainties for the total background predictions account for the correlations between the uncertainties of the different background contributions

Process	$m_{\ell \ell ^{\prime }}>600$ GeV	$m_{\ell \ell ^{\prime }}<600$ GeV
(a) $e\mu$ channel		
Top quark	1190 ± 140	22 ± 5
Diboson	159 ± 17	4.9 ± 0.9
Multi-jet and $W$+jets	55 ± 11	2.7 ± 1.7
$Z/\gamma ^{*}\rightarrow ll$	14.5 ± 2.0	0.18 ± 0.04
Total SM background	1410 ± 150	30 ± 7
SM+$Z^\prime$ ($M_{Z^\prime }=2$ TeV)	–	75 ± 13
SM+$\tilde{\nu }_{\tau }$ ($M_{\tilde{\nu }_{\tau }}=2$ TeV)	–	40 ± 8
SM+QBH RS $n = 1$ ($M_{\mathrm {th}}=2$ TeV)	–	44 ± 9
Data	1463	25
(b) $e\tau$ channel		
Top quark	790 ± 190	25 ± 9
Diboson	109 ± 26	6.2 ± 1.9
Multi-jet and $W$+jets	3200 ± 800	45 ± 14
$Z/\gamma ^{*}\rightarrow ll$	1030 ± 240	5.2 ± 1.4
Total SM background	5200 ± 1300	81 ± 25
SM+$Z^\prime$ ($M_{Z^\prime }=1.5$ TeV)	–	185 ± 34
SM+$\tilde{\nu }_{\tau }$ ($M_{\tilde{\nu }_{\tau }}=1.5$ TeV)	–	105 ± 27
SM+QBH RS $n = 1$ ($M_{\mathrm {th}}=1.5$ TeV)	–	122 ± 28
Data	5416	111
(c) $\mu \tau$ channel		
Top quark	580 ± 140	21 ± 7
Diboson	84 ± 20	4.8 ± 1.4
Multi-jet and $W$+jets	1900 ± 500	34 ± 12
$Z/\gamma ^{*}\rightarrow ll$	610 ± 140	2.6 ± 0.7
Total SM background	3200 ± 800	63 ± 20
SM+$Z^\prime$ ($M_{Z^\prime }=1.5$ TeV)	–	130 ± 28
SM+$\tilde{\nu }_{\tau }$ ($M_{\tilde{\nu }_{\tau }}=1.5$ TeV)	–	78 ± 22
SM+QBH RS $n = 1$ ($M_{\mathrm {th}}=1.5$ TeV)	–	90 ± 23
Data	3239	48

**Fig. 1:**
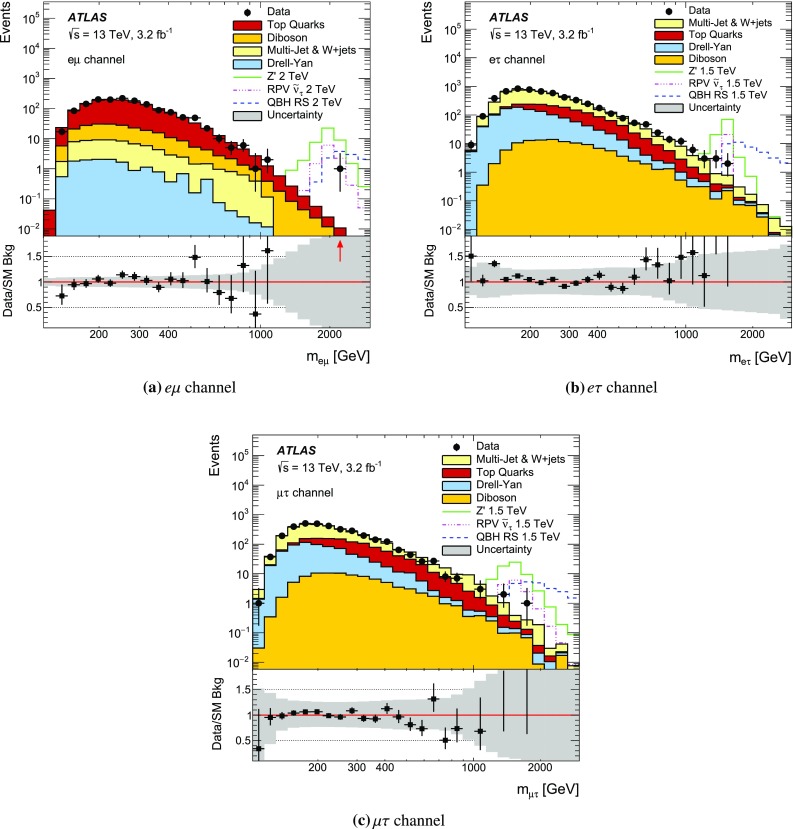
The invariant mass distribution of final selected. **a** $e\mu$, **b** $e\tau$ and **c** $\mu \tau$ pairs for data and MC predictions. Three selected signals are overlaid: a $Z^\prime$ with a mass of 2.0 and 1.5 TeV, a $\tau$ sneutrino ($\tilde{\nu }_{\tau }$) with a mass of 2.0 and 1.5 TeV, and a RS quantum black hole (QBH) with a threshold mass of 2.0 and 1.5 TeV. The signal mass point shown corresponds to the highest acceptance times efficiency in each channel. The *error bars* show the statistical uncertainty of the observed yields corresponding to a 68 % interval in a Poisson distribution, while the band in the *bottom plot* includes all systematic uncertainties added in quadrature

Figure 1 shows the $e\mu$, $e\tau$ and $\mu \tau$ invariant mass distribution. The event with the largest dilepton invariant mass is found in the $e\mu$ channel with $m_{e\mu }$=2.1  TeV. Since the SM expectation for $m_{e\mu }>$ 2 TeV is 0.02±0.02 events, the probability of observing one or more events is 2.6 %. It is then concluded that the observation of this high-mass candidate event is compatible with a statistical fluctuation and no significant excess is found over the expected background. Therefore, the observed data are concluded to be consistent with the SM prediction, and model-dependent exclusion limits are extracted using the techniques described in Sect. 7.

**Fig. 2:**
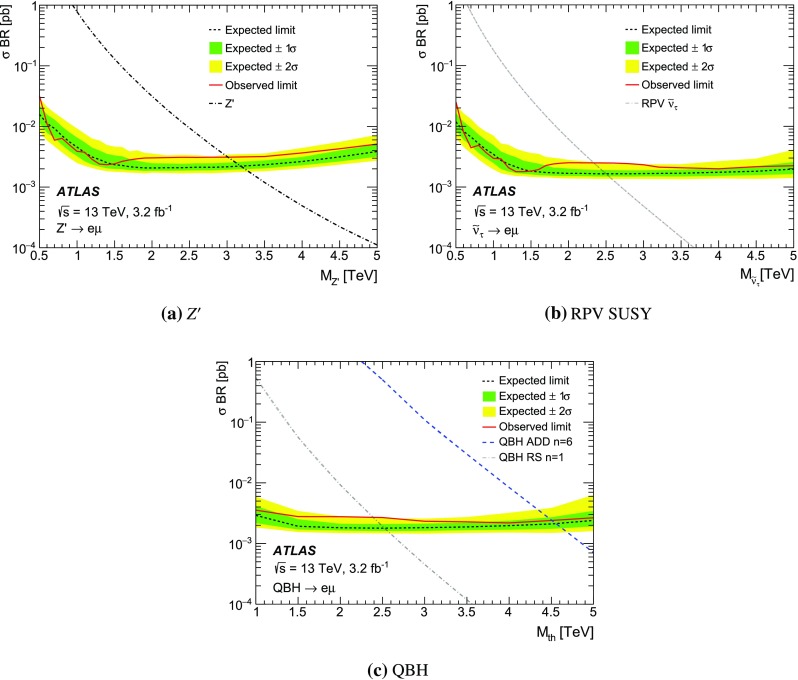
The observed and expected 95 % credibility level upper limits on the **a** $Z^\prime$, **b** $\tau$ sneutrino ($\tilde{\nu }_{\tau }$) and **c** QBH ADD and RS production cross-section times branching ratio in decays to an $e\mu$ final state. The signal theoretical cross-section times branching ratio lines for the $Z'$ model, the QBH ADD model assuming six extra dimensions and the RS model with one extra dimension are obtained from the Monte Carlo generators simulating each process, while the RPV SUSY $\tilde{\nu }_{\tau }$ includes the NLO *K*-factor calculated using LoopTools [38]. The expected limits are plotted with the ±1 and ±2 standard deviation uncertainty bands

**Fig. 3:**
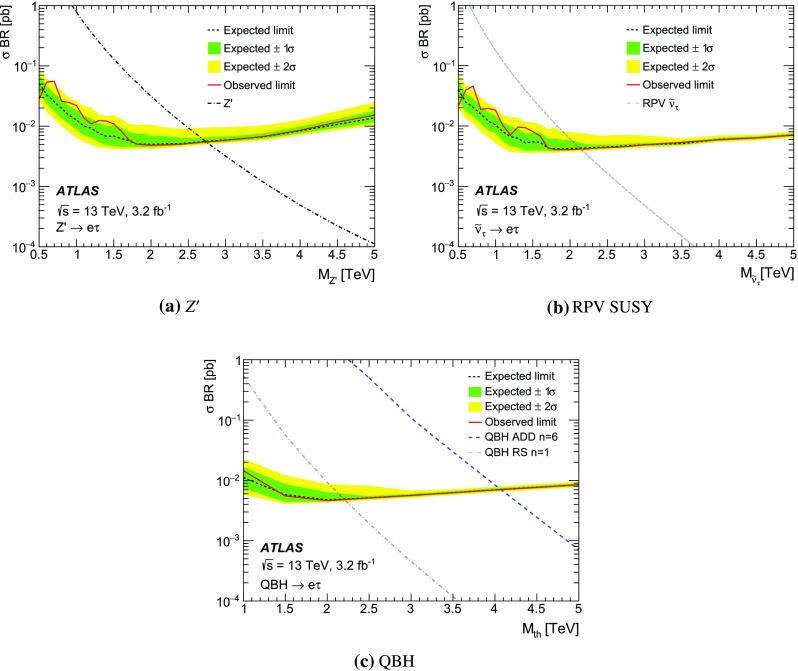
The observed and expected 95 % credibility level upper limits on the (a) $Z^\prime$, (b) $\tau$ sneutrino ($\tilde{\nu }_{\tau }$) and (c) QBH ADD and RS production cross-section times branching ratio in decays to an $e\tau$ final state. The signal theoretical cross-section times branching ratio lines for the $Z'$ model, the QBH ADD model assuming six extra dimensions and the RS model with one extra dimension are obtained from the Monte Carlo generators simulating each process, while the RPV SUSY $\tilde{\nu }_{\tau }$ includes the NLO *K*-factor calculated using LoopTools [38]. The expected limits are plotted with the $\pm 1$ and $\pm 2$ standard deviation uncertainty bands

**Fig. 4:**
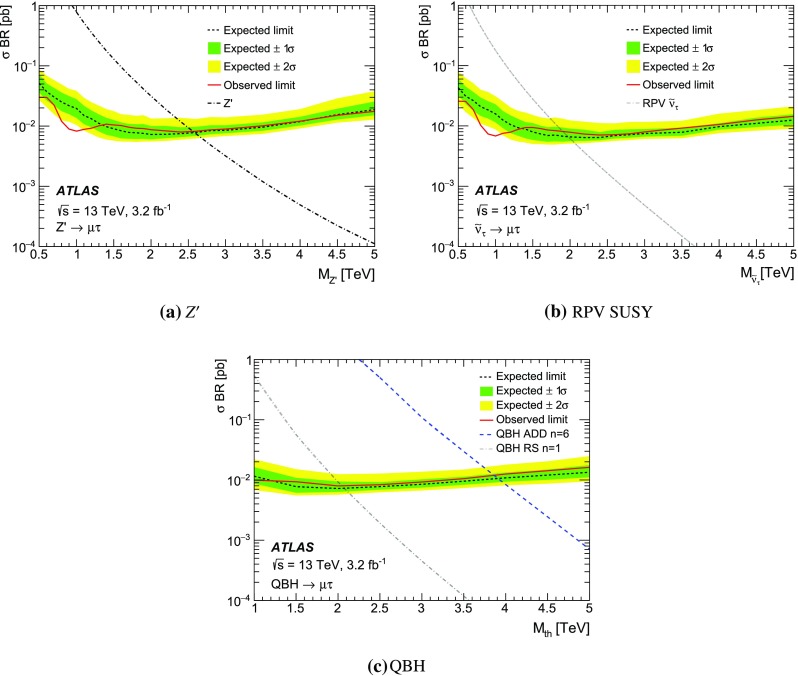
The observed and expected 95 % credibility level upper limits on the **a** $Z^\prime$, **b** $\tau$ sneutrino ($\tilde{\nu }_{\tau }$) and **c** QBH ADD and RS production cross-section times branching ratio in decays to an $\mu \tau$ final state. The signal theoretical cross-section times branching ratio lines for the $Z'$ model, the QBH ADD model assuming six extra dimensions and the RS model with one extra dimension are obtained from the Monte Carlo generators simulating each process, while the RPV SUSY $\tilde{\nu }_{\tau }$ includes the NLO *K*-factor calculated using LoopTools [38]. The expected limits are plotted with the $\pm 1$ and $\pm 2$ standard deviation uncertainty bands

Figures 2, 3 and 4 show the 95 % CL expected and observed upper limits on the production cross-section times branching ratio of the $Z^\prime$, RPV SUSY $\tilde{\nu }_{\tau }$ and QBH models for each of the final states considered. The extracted limits worsen for signal masses above 2.5 (1.5)  TeV in the $e\mu$ ($e\tau$ and $\mu \tau$) channel due to a decrease in the lepton reconstruction efficiency at very high $p_{\mathrm {T}}$. Results are summarised in Table 3. The $A\epsilon$ of the ADD and RS QBH models were found to agree within 1 % and therefore the same curve is used for the limit extraction.

**Table 3:** Expected and observed 95 % credibility level lower limits on the mass of a $Z^\prime$ with lepton-flavour-violating couplings, a supersymmetric $\tau$ sneutrino ($\tilde{\nu }_{\tau }$) with *R*-parity-violating couplings, and the threshold mass for quantum black hole production for the ADD $n=6$ and RS $n=1$ models. Limits for all channels are reported

Model	Expected limit (TeV)			Observed limit (TeV)		
	$e\mu$	$e\tau$	$\mu \tau$	$e\mu$	$e\tau$	$\mu \tau$
$Z^\prime$	3.2	2.7	2.6	3.0	2.7	2.6
RPV SUSY $\tilde{\nu }_{\tau }$	2.5	2.1	2.0	2.3	2.2	1.9
QBH ADD $n=6$	4.6	4.1	3.9	4.5	4.1	3.9
QBH RS $n=1$	2.5	2.2	2.1	2.4	2.2	2.1

## Conclusions

A search for a heavy particle decaying into an $e\mu$, $e\tau$ or $\mu \tau$ $(\ell \ell ^{\prime })$ final state is conducted, using 3.2 fb$^{-1}$ of $\sqrt{s}=13$ TeV proton–proton collision data recorded by the ATLAS detector at the Large Hadron Collider. The data are found to be consistent with the Standard Model prediction in both the validation region ($m_{\ell \ell ^{\prime }}<600$ GeV) and search region ($m_{\ell \ell ^{\prime }}>600$ GeV). With no evidence of new physics, Bayesian lower limits at 95 % credibility level are set on the mass of a $Z^\prime$ vector boson with lepton-flavour-violating couplings at 3.0, 2.7 and 2.6 TeV separately for $e\mu$, $e\tau$ and $\mu \tau$ pairs, and a supersymmetric $\tau$ sneutrino ($\tilde{\nu }_{\tau }$) with *R*-parity-violating couplings at 2.3, 2.2 and 1.9 TeV. The results are also interpreted as limits on the threshold mass for quantum black hole production. The exclusion limits extracted on the mass of a $Z^\prime$ and the supersymmetric $\tau$ sneutrino extend by around 20 % those reported by ATLAS and CMS using the full dataset at $\sqrt{s}$ = 8 TeV.

## Footnotes

1 ATLAS uses a right-handed coordinate system with its origin at the nominal interaction point (IP) in the centre of the detector and the *z*-axis along the beam pipe. The *x*-axis points from the IP to the centre of the LHC ring, and the *y*-axis points upward. The *x*–*y* plane is referred to as the transverse plane, used to define quantities such as the transverse momentum ($p_{\mathrm {T}}$). Cylindrical coordinates $(r,\phi )$ are used in the transverse plane, $\phi$ being the azimuthal angle around the *z*-axis. The pseudorapidity is defined in terms of the polar angle $\theta$ as $\eta =-\ln \tan (\theta /2)$. Angular distance is measured in units of $\Delta R = \sqrt{ (\Delta \eta )^{2} + (\Delta \phi )^{2} }$.

2 For the $\mu \tau$ channel, the muon acceptance is limited by the coverage of the muon trigger system ($|\eta |<2.4$).

3 The primary vertex corresponds to the interaction vertex with the highest $p_{\mathrm {T}}^2$ sum of all tracks belonging to it.

4 A loose lepton is defined as a lepton satisfying all requirements except isolation for muons and a looser identification requirement (*LH-Medium*) for electrons. No loose $\tau$ lepton category is defined.

5 The acceptance (*A*) defines the geometrical and kinematic region covered by the detector. The efficiency ($\epsilon$) is the fraction of events falling in the detector acceptance region that fulfil all selection criteria. Therefore, $A\epsilon$ is the fraction of events that pass all the selection requirements.
